# Discrete-Modulated Coherent-State Quantum Key Distribution with Basis-Encoding

**DOI:** 10.34133/research.0691

**Published:** 2025-05-14

**Authors:** Mingxuan Guo, Peng Huang, Le Huang, Xiaojuan Liao, Xueqin Jiang, Tao Wang, Guihua Zeng

**Affiliations:** ^1^ State Key Laboratory of Photonics and Communications, Institute for Quantum Sensing and Information Processing, Shanghai Jiao Tong University, Shanghai 200240, China.; ^2^ Shanghai Research Center for Quantum Sciences, Shanghai 201315, China.; ^3^ Hefei National Laboratory, Hefei 230088, China.; ^4^ College of Information Science and Technology, Donghua University, Shanghai 201620, China.; ^5^ Shanghai XunTai Quantech Co., Ltd, Shanghai, 200241, China.

## Abstract

Discrete-modulated coherent-state continuous-variable quantum key distribution (DMCS-CVQKD) is of great value for its simple implementation. However, the traditional DMCS-CVQKD scheme cannot tolerate the high channel excess noise and channel loss, compared to the Gaussian-modulated scheme, and its error correction is still difficult. In this paper, we propose a discrete-modulated coherent-state basis-encoding quantum key distribution (DMCS-BE-QKD) protocol, where the secret keys are encoded in the random choice of 2 measurement bases, i.e., the conjugate quadratures *X* and *P* of discrete-modulated coherent states, and it only needs simple binary sequence error correction. We analyze the secret key rate of DMCS-BE-QKD protocol under individual and collective attacks in the linear Gaussian channel. The results show that DMCS-BE-QKD can greatly enhance the ability to tolerate the channel loss and excess noise compared to the original DMCS-CVQKD protocol, which can tolerate approximately 40 dB more channel loss compared to the original DMCS-CVQKD for the realistic value of noise. Finally, a proof-of-principle experiment is conducted under a 50.5-km optical fiber to verify the feasibility of DMCS-BE-QKD. It is based on the consistent physical procedures of the traditional DMCS-CVQKD, which makes it perfectly compatible to deployed terminals and can serve as a multiplier for the practical secure quantum cryptography communication in harsh environments.

## Introduction

Continuous-variable quantum key distribution (CVQKD) can enable remote trusted parties to share secure secret keys through the untrusted quantum channel with the coherent source and detection, which can be categorized into 2 families, i.e., the Gaussian-modulated coherent-state (GMCS)-CVQKD [1–5] and discrete-modulated coherent-state (DMCS)-CVQKD [6–8] protocols. For the former one, coherent states are modulated according to a Gaussian distribution with an infinite-size constellation. For the latter one, coherent states are modulated according to a discrete probability distribution with a finite-size constellation. The key information is commonly encoded in random amplitudes and phases of coherent states. So far, the security of certain GMCS-CVQKD and DMCS-CVQKD protocols have been proved against individual [9–12], collective [13–19], and coherent attacks [20–23] even when taking into account the finite-size effect [24–29]. Moreover, certain protocols have been experimentally realized in both laboratory [30–35] and field tests [36–39], which show its superior applicability in metropolitan area quantum networks.

As known, the implementations of the above CVQKD protocols, especially the DMCS ones, are well compatible with classical coherent optical communication infrastructures [40]. Moreover, compared to GMCS-CVQKD, DMCS-CVQKD has lower requirements for modulation devices. However, as a cost, DMCS-CVQKD has a low tolerance for the channel loss and the excess noise. Moreover, as far as error correction is concerned, the error correction of conventional DMCS-CVQKD is still similar to that of GMCS-CVQKD, which is difficult and complex to implement, because the values of DMCS-CVQKD’s measurement results are continuous. In addition, the DMCS-CVQKD transmits lower power, which corresponds to lower signal-to-noise ratio. Therefore, error correction of conventional DMCS-CVQKD remains difficult.

Huang et al. [41] report a novel protocol with basis-encoding (BE) of Gaussian-modulated coherent states, where the key information is encoded in the random choice of 2 measurement bases, i.e., the choice of conjugate quadratures *X* and *P*. This encoding method is different from the encoding method for conventional CVQKD protocols, which encodes the key in the value of conjugate quadratures *X* and *P* of the quantum state. The BE method exhibits the higher tolerable excess noise against the typical non-Gaussian individual attack as explored in [41]. Moreover, the raw keys of both Alice and Bob in the BE scheme are binary sequences, and the error correction for them is easier to perform compared to traditional CVQKD protocols. However, this work only analyzes the secret key rate of BE-QKD under a typical non-Gaussian individual attack. It has marked limitations since the Gaussian channel (i.e., Gaussian attack) is the more general case in reality. Meanwhile, it does not analyze the secret key rate of BE-QKD under collective attacks. No experiment is conducted to verify the feasibility of BE-QKD. Since the publication of [41], the security analysis [16–19,27–29] and experiments [34,35,42–44] of the conventional CVQKD have made significant progress. However, the development of BE-QKD has been rather limited.

Inspired by this, a discrete-modulated coherent-state basis-encoding quantum key distribution (DMCS-BE-QKD) protocol is proposed to improve the tolerance of DMCS-CVQKD for the channel loss and excess noise, and the security analysis of it is further improved compared to the former work [41], where a novel security analysis framework is developed because the analysis framework in [41] is not suitable for analyzing the secret key rate under Gaussian individual or collective attacks. We first focus on 2 typical DMCS-BE-QKD protocols: binary-phase-shift-keying (BPSK) BE-QKD and quadrature-phase-shift-keying (QPSK) BE-QKD protocols. We develop the methods to evaluate the secret key rate of B/QPSK-BE-QKD in the linear Gaussian channel under individual attacks and collective attacks. Moreover, similar analysis can be extended to the arbitrary modulation case. The simulation result shows that B/QPSK-BE-QKD can significantly improve the transmission distance compared to the original B/QPSK-CVQKD scheme and QPSK-BE-QKD performs better than BPSK-BE-QKD. Translating the whole constellation diagram along the y=x axis does not affect the key rate due to the fact that the probability density curves of measurement results corresponding to perform *X* and *P* measurements are always the same in this case. Moreover, we realize the proof-of-principle experiment of QPSK-BE-QKD under a 50.5-km optical fiber with the 11-dB channel loss.

## Results

### DMCS-BE-QKD protocol

The DMCS-BE-QKD protocol executes the following steps.

Quantum communication part:

1. Alice randomly prepares coherent states $|\alpha_{k}\rangle =$ $|ℜ\left(\alpha_{k}\right)+\mathrm{iℑ}\left(\alpha_{k}\right)\rangle$ from the set ${\left\{|\alpha_{k}\rangle \right\}}_{k=0,\ldots ,M-1}$, where $\alpha_{k}\in \mathbb{C}$ and *M* represents the modulation order. Then, Alice sends them to Bob;
2. Bob randomly chooses a random binary sequence *b* to decide the measurement basis, i.e., quadrature *X* (corresponding to *b* = 0) or *P* (corresponding to *b* = 1), to measure and obtain measurement results $X_{B}$ or $P_{B}$ (shot noise unit);
3. Alice and Bob randomly choose a fraction of measurement results to perform the parameter estimation, including the modulation variance, excess noise, and the transmission efficiency.

Key decoding and distillation part:

4. Bob publishes his measurement outcomes $\beta_{y}$ of his homodyne detection, i.e., the values $X_{B}$ or $P_{B}$ and Alice decoding the secret key *b* by judging the Bob’s measurement basis according to the decoding rules related with her generated coherent states. After these operations, Alice and Bob share a set of correlated binary raw keys.
5. Alice and Bob perform the reconciliation with binary codes and the privacy amplification to distill final secret keys. Intuitively, Bob can also apply the heterodyne detection and publishes randomly one of the 2 basis outcomes according to *b* = 0 or 1.

**Fig. 1.**
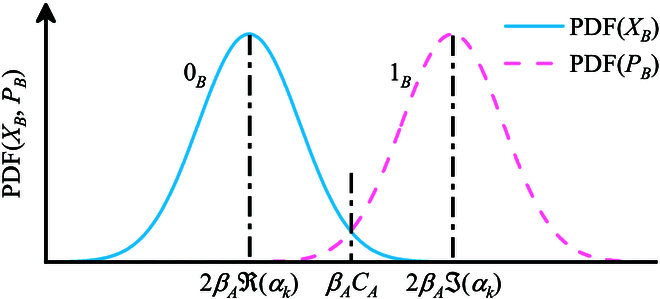
The decoding rules for Alice. Alice decodes the key based on how “close” is the measurement result $\beta_{y}$ to $2\beta_{A}ℜ\left(\alpha_{k}\right)$ and $2\beta_{A}ℑ\left(\alpha_{k}\right)$. When $\beta_{y}$ is closer to $2\beta_{A}ℜ\left(\alpha_{k}\right)$, Alice decodes the key as 0. Otherwise, Alice decodes the key as 1.

As depicted in Fig. 1, the decoding rules in step (4) are summarized as follows:

when $ℜ\left(\alpha_{k}\right)>ℑ\left(\alpha_{k}\right)$ and $\beta_{y}>\beta_{A}C_{A}$, decode key as 0

when $ℜ\left(\alpha_{k}\right)>ℑ\left(\alpha_{k}\right)$ and $\beta_{y}<\beta_{A}C_{A}$, decode key as 1

when $ℜ\left(\alpha_{k}\right)<ℑ\left(\alpha_{k}\right)$ and $\beta_{y}>\beta_{A}C_{A}$, decode key as 1

when $ℜ\left(\alpha_{k}\right)<ℑ\left(\alpha_{k}\right)$ and $\beta_{y}<\beta_{A}C_{A}$, decode key as 0

where $C_{A}=\frac{1}{2}\left(2ℜ\left(\alpha_{k}\right)+2ℑ\left(\alpha_{k}\right)\right)$, and $\beta_{A}$ is the coefficients to give minimum variances of $\Delta X=X_{B}-\beta_{A}X_{A}$ and $\Delta P=P_{B}-\beta_{A}P_{A}$. When Bob uses the homodyne detector, $\beta_{A}=\sqrt{\mathrm{T\eta}}$, where *T* is the channel trasmission efficiency and η is the efficiency of the detection. When Bob uses the heterodyne detector, $\beta_{A}=\sqrt{\mathrm{T\eta}/2}$. The basic principle of decoding is finding the smaller distance from the Bob’s measurement result to the original values of conjugate quadratures. We further discuss several possible situations when Alice conducts the correct and incorrect decoding.

Correct decoding:

when $ℜ\left(\alpha_{k}\right)>ℑ\left(\alpha_{k}\right)$, $X_{B}>\beta_{A}C_{A}$ or $P_{B}<\beta_{A}C_{A}$

when $ℜ\left(\alpha_{k}\right)<ℑ\left(\alpha_{k}\right)$, $X_{B}<\beta_{A}C_{A}$ or $P_{B}>\beta_{A}C_{A}$

Incorrect decoding:

when $ℜ\left(\alpha_{k}\right)>ℑ\left(\alpha_{k}\right)$, $X_{B}<\beta_{A}C_{A}$ or $P_{B}>\beta_{A}C_{A}$

when $ℜ\left(\alpha_{k}\right)<ℑ\left(\alpha_{k}\right)$, $X_{B}>\beta_{A}C_{A}$ or $P_{B}<\beta_{A}C_{A}$

It should be mentioned that the quantum communication part works the same as the traditional DMCS-CVQKD protocol, while the key decoding and distillation parts run differently.

### The security against individual and collective attacks with B/QPSK modulation

We divide the overall channel into subchannels, based on Bob’s measurement result $\beta_{y}$. Each subchannel is defined by a specific measurement result $\beta_{y}$. Then, we can analyze the security of each subchannel to obtain its secret key rate. The secret key rates of subchannels are weighted according to the probability of the subchannel occurrence and summed to obtain the final secret key rate. We can use the probability theory to calculate the classical mutual information between Alice and Bob in the subchannel. For Eve, in the subchannel $\beta_{y}=m$, the quantum state she obtains when Bob encodes 0 is $\rho_{E}^{x=m}$, and the quantum state she obtains when Bob encodes 1 is $\rho_{E}^{p=m}$. Using the density matrix $\rho_{E}^{x=m}$ and $\rho_{E}^{p=m}$, we can determine the maximum information Eve can obtain in each subchannel. Then, we can easily calcualte the secret key rate of each subchannel and further obtain the overall key rate.

For each subchannel $\beta_{y}=m$ (including $X_{B}=m$ and $P_{B}=m$), the secret key rate of BE-QKD under individual attacks is given by

$$\begin{array}{c} K_{\mathrm{ind}}^{\beta_{y}=m}=\beta I\left(A;\;B|\beta_{y}=m\right)-\underset{\Pi }{\text{max}}I\left(b;\;E,\;\Pi |\beta_{y}=m\right) \end{array}$$ (1)

where Π represents any group of Eve’s positive-operator valued measurements, $\Pi =\left\{\Pi_{1},\;\Pi_{2},\ldots \right\}$. For the subchannel, the secret key rate of BE-QKD under collective attacks is given by [45],

$$K_{\mathrm{col}}^{\beta_{y}=m}=\beta I\left(A;\;B|\beta_{y}=m\right)-\chi_{\mathrm{bE}}^{\beta_{y}=m}$$ (2)

$$\begin{array}{c} \chi_{\mathrm{bE}}^{\beta_{y}=m}=\\ S\left(\rho_{E}^{\beta_{y}=m}\right)-p\left(0_{B}|\beta_{y}=m\right)S\left(\rho_{E}^{x=m}\right)-p\left(1_{B}|\beta_{y}=m\right)S\left(\rho_{E}^{p=m}\right) \end{array}$$ (3)

where $\chi_{\mathrm{bE}}$ represents the Holevo bound, the density matrix of the state at *E* in the subchannel $\beta_{y}=m$ is $\begin{array}{c} \rho_{E}^{\beta_{y}=m}=p\left(0_{B}|\beta_{y}=m\right) \end{array}$$\begin{array}{c} \rho_{E}^{x=m}+p\left(1_{B}|\beta_{y}=m\right)\rho_{E}^{p=m} \end{array}$, “$0_{B}$” represents Bob encodes her key as 0, and “$1_{B}$” represents Bob encodes her key as 1.

So, the overall key rate of BE-QKD under individual and collective attacks is given by,

$$K_{\mathrm{ind}}=\int p\left(\beta_{y}=m\right)K_{\mathrm{ind}}^{\beta_{y}=m}\mathrm{dm}$$ (4)

$$K_{\mathrm{col}}=\int p\left(\beta_{y}=m\right)K_{\mathrm{col}}^{\beta_{y}=m}\mathrm{dm}$$ (5)

**Fig. 2.**
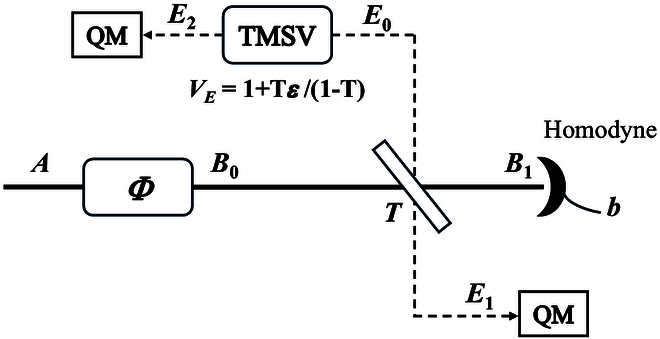
The operation principle of entangling cloner attacks when Bob uses the homodyne detection while ignoring the detection efficiency and electrical noise.

We will now discuss the secret key rate of B/QPSK-BE-QKD in the linear Gaussian channel. In the linear Gaussian channel, the channel loss is the coefficient *T* (i.e., the channel loss can be fully characterized by the coefficient *T*), and the noise is the additive Gaussian noise that is independent of the input data. The linear Gaussian channel is the most common and important channel in reality, where the attack from Eve is entangling cloner attack [12]. So, we need to discuss the security against individual and collective entangling cloner attacks. The entangling cloner attack is depicted in Fig. 2. Eve first prepares a 2-mode squeezed state (TMSV) with variance $V_{E}=1+\frac{\mathrm{T\varepsilon}}{1-T}$, where ε represents the channel excess noise. Then, Eve reserves one mode of TMSV $E_{2}$ and coupling another mode $E_{0}$ with Alice’s outgoing signal $B_{0}$ in a beam splitter with transmissivity *T*. One of the outgoing modes of the beam splitter $B_{1}$ is sent to Bob through a lossless channel, and another mode $E_{1}$ is reserved by Eve. Finally, Eve performs optimal measurements on the mode $E_{1}E_{2}$.

#### Mutual information between Alice and Bob

**Fig. 3.**
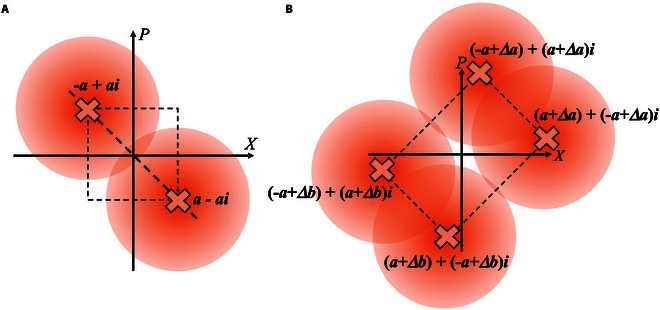
The modulation constellation for (A) BPSK-BE-QKD and (B) QPSK-BE-QKD.

We first discuss the mutual information between Alice and Bob for BPSK-BE-QKD. Here, we discuss the simplest situation, i.e., the modulation constellation ($\alpha_{0}=-a+\mathrm{ai}$, $\alpha_{1}=a-\mathrm{ai}$) shown as Fig. 3A is used, Bob uses the homodyne detection, and the detection efficiency and the electrical noise are ignored. The more complex situations and calculation detail are discussed in Supplementary Note I. First, we can write down the conditional probabilities of Bob’s measurement results,

$$\begin{array}{c} \begin{array}{l} p\left(\beta_{y}=m|\alpha_{0},\;0_{B}\right)=N_{\mathrm{pdf}}\left(m,\;-2\sqrt{T}a,\;1+\mathrm{T\varepsilon}\right)=p_{1},\\ p\left(\beta_{y}=m|\alpha_{1},\;0_{B}\right)=N_{\mathrm{pdf}}\left(m,\;2\sqrt{T}a,\;1+\mathrm{T\varepsilon}\right)=p_{2},\\ p\left(\beta_{y}=m|\alpha_{0},\;1_{B}\right)=N_{\mathrm{pdf}}\left(m,\;2\sqrt{T}a,\;1+\mathrm{T\varepsilon}\right)=p_{2},\\ p\left(\beta_{y}=m|\alpha_{1},\;1_{B}\right)=N_{\mathrm{pdf}}\left(m,\;-2\sqrt{T}a,\;1+\mathrm{T\varepsilon}\right)=p_{1}, \end{array} \end{array}$$ (6)

where $N_{\mathrm{pdf}}\left(•,\;\mu ,\;\sigma^{2}\right)$ represents the probability density function of the normal distribution with the mean value μ and the standard variance σ. We can also calculate the conditional error rate $p\left(1_{A}|0_{B},\;\beta_{y}=m\right)$ when Bob encodes the key “0” and $p\left(0_{A}|1_{B},\;\beta_{y}=m\right)$ when Bob encodes the key “1”,

$$\begin{array}{c} p\left(1_{A}|0_{B},\;\beta_{y}=m\right)=\\ p\left(0_{A}|1_{B},\;\beta_{y}=m\right)=p_{\text{error}}^{\text{BPSK}}=1/\left(1+\text{exp},\left|\frac{4\sqrt{T}\mathrm{am}}{1+\mathrm{T\varepsilon}}\right|,\right) \end{array}$$ (7)

Above all, the mutual information between Alice and Bob for BPSK-BE-QKD is given by,

$$\begin{array}{c} I\left(A;\;B|\beta_{y}=m\right)=\\ H\left(A|\beta_{y}=m\right)-H\left(A|B,\;\beta_{y}=m\right)=1-H\left(p_{\text{error}}^{\text{BPSK}}\right). \end{array}$$ (8)

Then, we discuss the mutual information between Alice and Bob for QPSK-BE-QKD. We discuss the situation that the modulation constellation ($\alpha_{0}=-a+\Delta a+\left(a+\Delta a\right)i$, $\Delta b+\alpha_{1}=-a+$$\left(a+\Delta b\right)i$, $\alpha_{2}=a+\Delta b+\left(-a+\Delta b\right)i$, $\alpha_{3}=a+$ $\Delta a+\left(-a+\Delta a\right)i$, Δa>Δb) shown as Fig. 3B is used, Bob uses the homodyne detection, and the detection efficiency and the electrical noise are ignored. The more complex situations and calculation detail are discussed in Supplementary Note I. We can write down the conditional probabilities of Bob’s measurement results,

$$\begin{array}{c} \begin{array}{c} p\left(\beta_{y}=m\left|\alpha_{0},\;0_{B}\right.\right)=N_{\mathrm{pdf}}\left(m,\;2\sqrt{T}\left(-\alpha +∆\alpha \right),\;1+\mathrm{T\varepsilon}\right)=p_{1}\\ p\left(\beta_{y}=m\left|\alpha_{1},\;0_{B}\right.\right)=N_{\mathrm{pdf}}\left(m,\;2\sqrt{T}\left(-\alpha +∆b\right),\;1+\mathrm{T\varepsilon}\right)=p_{2}\\ \begin{array}{c} p\left(\beta_{y}=m\left|\alpha_{2},\;0_{B}\right.\right)=N_{\mathrm{pdf}}\left(m,\;2\sqrt{T}\left(\alpha +∆b\right),\;1+\mathrm{T\varepsilon}\right)=p_{3}\\ p\left(\beta_{y}=m\left|\alpha_{3},\;0_{B}\right.\right)=N_{\mathrm{pdf}}\left(m,\;2\sqrt{T}\left(\alpha +∆\alpha \right),\;1+\mathrm{T\varepsilon}\right)=p_{4}\\ \begin{array}{c} p\left(\beta_{y}=m\left|\alpha_{0},\;1_{B}\right.\right)=N_{\mathrm{pdf}}\left(m,\;2\sqrt{T}\left(\alpha +∆\alpha \right),\;1+\mathrm{T\varepsilon}\right)=p_{4}\\ p\left(\beta_{y}=m\left|\alpha_{1},\;1_{B}\right.\right)=N_{\mathrm{pdf}}\left(m,\;2\sqrt{T}\left(\alpha +∆b\right),\;1+\mathrm{T\varepsilon}\right)=p_{3}\\ \begin{array}{c} p\left(\beta_{y}=m\left|\alpha_{2},\;1_{B}\right.\right)=N_{\mathrm{pdf}}\left(m,\;2\sqrt{T}\left(-\alpha +∆b\right),\;1+\mathrm{T\varepsilon}\right)=p_{2}\\ p\left(\beta_{y}=m\left|\alpha_{3},\;1_{B}\right.\right)=N_{\mathrm{pdf}}\left(m,\;2\sqrt{T}\left(-\alpha +∆\alpha \right),\;1+\mathrm{T\varepsilon}\right)=p_{1}. \end{array} \end{array} \end{array} \end{array} \end{array}$$ (9)

Similarly, we can also give out the conditional error rate $p\left(1_{A}|0_{B},\;\beta_{y}=m\right)$ and $p\left(0_{A}|1_{B},\;\beta_{y}=m\right)$ for QPSK-BE-QKD,

$$\begin{array}{c} p\left(1_{A}|0_{B},\;\beta_{y}=m\right)=p\left(0_{A}|1_{B},\;\beta_{y}=m\right)=p_{\text{error}}^{\text{QPSK}}=\\ \left\{\begin{array}{cc} & \frac{p_{1}+p_{2}}{p_{1}+p_{2}+p_{3}+p_{4}},m>2\sqrt{T}\Delta a\\ & \frac{p_{2}+p_{4}}{p_{1}+p_{2}+p_{3}+p_{4}},2\sqrt{T}\Delta b<m<2\sqrt{T}\Delta a\\ & \frac{p_{3}+p_{4}}{p_{1}+p_{2}+p_{3}+p_{4}},m<2\sqrt{T}\Delta b \end{array}\right. \end{array}$$ (10)

Above all, the mutual information between Alice and Bob for QPSK-BE-QKD is given by,

$$\begin{array}{c} I\left(A;\;B|\beta_{y}=m\right)=\\ H\left(A|\beta_{y}=m\right)-H\left(A|B,\;\beta_{y}=m\right)=1-H\left(p_{\text{error}}^{\text{QPSK}}\right) \end{array}$$ (11)

#### Leaked key information to Eve

In this part, we try to calculate the leaked key information to Eve in the linear Gaussian channel under individual attacks $\left(\;\underset{\Pi }{\text{max}}I\left(b;\;E,\;\Pi |\beta_{y}=m\right)\;\right)$ and under collective attacks $\left(\;\chi_{\mathrm{bE}}^{\beta_{y}=m}\;\right)$. In order to achieve this, we first need to figure out the density matrix of the conditional quantum state $\rho_{E}^{x=m}$ and $\rho_{E}^{p=m}$ when using the homodyne detection and ignoring the detection efficiency and the electrical noise.

We can first express the state at $B_{0}$ and two-mode squeezed vacuum state (TSMV) state $|E_{0}E_{2}\rangle$:

$$\rho_{B_{0}}=\sum_{k=0}^{M-1}p_{k}|\alpha_{k}\rangle \;\langle \alpha_{k}|$$ (12)

$$\begin{array}{c} |E_{0}E_{2}\rangle =\frac{1}{\text{cosh}\;r_{E}}\sum_{n=0}^{\infty }{\left(\text{tanh}\;r_{E}\right)}^{n}|n,\;n\rangle , \end{array}$$ (13)

where $r_{E}=\left({\text{cosh}}^{-1}V_{E}\right)\;/\;2$ and *M* = 2 or 4 when the protocol is B/QPSK-BE-QKD protocol. The operator of the beam splitter with transmissivity *T* can be given by [46],

$$\begin{array}{c} R_{B_{0}E_{0}}=\text{exp}\left({\text{cos}}^{-1}\sqrt{T}\left({\hat{a}}_{B_{0}}⊗{\hat{a}}_{E_{0}}^{†}-\;{\hat{a}}_{B_{0}}^{†}⊗{\hat{a}}_{E_{0}}\right)\right) \end{array}$$ (14)

Then, we can calculate the density matrix of the state at $B_{1}E_{1}E_{2}$,

$$\rho_{B_{1}E_{1}E_{2}}=\left(R_{B_{0}E_{0}}⊗I_{E_{2}}\right)\left(\rho_{B_{0}}⊗|E_{0}E_{2}\rangle \;\langle E_{0}E_{2}|\right)\left(R_{B_{0}E_{0}}^{†}⊗I_{E_{2}}\right)$$ (15)

We define eigenvalue sets of the $\hat{x}$ and $\hat{p}$ operator [the definitions of $\hat{x}$ and $\hat{p}$ are $\hat{x}=\hat{a}+{\hat{a}}^{†}$ and $\hat{p}=i\left({\hat{a}}^{†}-\hat{a}\right)$, where ${\hat{a}}^{†}$ is the creation operator and $\hat{a}$ is the annihilation operator] as $\Lambda_{x}$ and $\Lambda_{p}$, respectively. To get numerical results, we cut off the space of $B_{i}$ and $E_{i}$ by the space spanned by the first *N* Fock states. Thus, we know that these 2 eigenvalue sets are completely the same and both of them have the size *N*. So, we define that $\Lambda =\Lambda_{x}=\Lambda_{p}=\left\{\lambda_{1},\ldots ,\lambda_{N}\right\}$, $\hat{x}|x_{\lambda_{i}}\rangle =\lambda_{i}|x_{\lambda_{i}}\rangle$, and $\hat{p}|p_{\lambda_{i}}\rangle =\lambda_{i}|p_{\lambda_{i}}\rangle$. For convenience, we abbreviate $|x_{\lambda_{i}}\rangle$ as $|x_{i}\rangle$ and abbreviate $|p_{\lambda_{i}}\rangle$ as $|p_{i}\rangle$. Then, we can define that,

$$M_{x=\lambda_{i}}=M_{x_{i}}=\left(|x_{i}\rangle \;\langle x_{i}|\right)⊗I_{E_{1}E_{2}}$$ (16)

$$M_{p=\lambda_{i}}=M_{p_{i}}=\left(|p_{i}\rangle \;\langle p_{i}|\right)⊗I_{E_{1}E_{2}}$$ (17)

Furthermore, we can calculate conditional probabilities of each measurement results and the probability of the subchannel occurrence,

$$p\left(\beta_{y}=\lambda_{i}|0_{B}\right)=\mathrm{Tr}\left(M_{x_{i}}^{†}M_{x_{i}}\rho_{B_{1}E_{1}E_{2}}\right)$$ (18)

$$p\left(\beta_{y}=\lambda_{i}|1_{B}\right)=\mathrm{Tr}\left(M_{p_{i}}^{†}M_{p_{i}}\rho_{B_{1}E_{1}E_{2}}\right)$$ (19)

$$p\left(\beta_{y}=\lambda_{i}\right)=p\left(0_{B}\right)p\left(\beta_{y}=\lambda_{i}|0_{B}\right)+p\left(1_{B}\right)p\left(\beta_{y}=\lambda_{i}|1_{B}\right)$$ (20)

Then, we give out the density matrix of the conditional quantum state $\rho_{E}^{x=\lambda_{i}}$ and $\rho_{E}^{p=\lambda_{i}}$,

$$\rho_{E}^{x=\lambda_{i}}=\rho_{E_{1}E_{2}}^{x=\lambda_{i}}={\mathrm{Tr}}_{B_{1}}\frac{M_{x_{i}}\rho_{B_{1}E_{1}E_{2}}M_{x_{i}}^{†}}{p\left(\beta_{y}=\lambda_{i}|0_{B}\right)}$$ (21)

$$\rho_{E}^{p=\lambda_{i}}=\rho_{E_{1}E_{2}}^{p=\lambda_{i}}={\mathrm{Tr}}_{B_{1}}\frac{M_{p_{i}}\rho_{B_{1}E_{1}E_{2}}M_{p_{i}}^{†}}{p\left(\beta_{y}=\lambda_{i}|1_{B}\right)}$$ (22)

The calculation methods for the density matrixes of the states $\rho_{E}^{x=\lambda_{i}}$ and $\rho_{E}^{p=\lambda_{i}}$ when using the heterodyne detection and in the cases with the imperfect detection are shown in Supplementary Note II. We can find that the calculation process involves the calculation about 3 state spaces, which will largely slow down our calculation speed. To solve this, we can derive the specific expression of $\rho_{E}^{x=\lambda_{i}}$ and $\rho_{E}^{p=\lambda_{i}}$ involving only about 2 state spaces to accelerate the speed for calculating, which is detailed in Supplementary Note III.

After we obtain the density matrixes of the states $\rho_{E}^{x=\lambda_{i}}$ and $\rho_{E}^{p=\lambda_{i}}$, we can calculate the leaked key information to Eve in the linear Gaussian channel under individual attacks and under collective attacks. Under individual attacks, we can use a non-convex optimization method [47] to calculate the $\underset{\Pi }{\text{max}}I\left(b;\;E,\;\Pi |\beta_{y}=\lambda_{i}\right)$ since $\underset{\Pi }{\text{max}}I\left(b;\;E,\;\Pi |\beta_{y}=\lambda_{i}\right)$ is a convex function and finding its maximum value is a non-convex optimization problem. The detail is described in Supplementary Note IV. Then, we can use Eqs. 1, 4, 6 to 8, and 12 to 22 to calculate the key rate for BPSK-BE-QKD and use Eqs. 1, 4, and 9 to 22 for QPSK-BE-QKD under individual attacks. Under collective attacks, we just need to calculate the related von Neumann entropy, which is easy to be calculated. Thus, we can use Eqs. 2, 3, 5 to 8, and 12 to 22 to calculate the key rate for BPSK-BE-QKD and use Eqs. 2, 3, 5, and 9 to 22 for QPSK-BE-QKD under collective attacks. It is worth noting that under the condition of the photon number cutoff, Eqs. 4 and 5 need to be rewritten as follows,

$$K_{\mathrm{ind}}=\int p\left(\beta_{y}=m\right)K_{\mathrm{ind}}^{\beta_{y}=m}\mathrm{dm}\approx \sum_{i=1}^{N}p\left(\beta_{y}=\lambda_{i}\right)K_{\mathrm{ind}}^{\beta_{y}=\lambda_{i}}$$ (23)

$$K_{\mathrm{col}}=\int p\left(\beta_{y}=m\right)K_{\mathrm{col}}^{\beta_{y}=m}\mathrm{dm}\approx \sum_{i=1}^{N}p\left(\beta_{y}=\lambda_{i}\right)K_{\mathrm{col}}^{\beta_{y}=\lambda_{i}}$$ (24)

### The security against individual and collective attacks with an arbitrary modulation

In this section, we discuss the secret key rate of DMCS-BE-QKD in the linear Gaussian channel under individual and collective attacks with an arbitrary modulation. A modulation with *M* coherent state ${\left\{|\alpha_{k}\rangle \right\}}_{k=0,..,M-1}$ is prepared with probabilities ${\left\{p_{k}\right\}}_{k=0,\ldots ,M-1}$. Then, we calculate the average value of real and imagine parts for coherent states and sort them from small to large to form the set $ϒ={\left\{\Delta_{k}^{C}\right\}}_{k=0,\ldots ,M_{1}-1}$ while removing the same elements (thus, $M_{1}\leq M$), where $\Delta_{k}^{C}=\frac{1}{2}\left(ℜ\left(\alpha_{k}\right)+ℑ\left(\alpha_{k}\right)\right)$. We also define the set $K_{x>p}$ that includes all the indices whose corresponding coherent states’ *X* value is larger than *P* value. We define the set $K_{x<p}$ that includes all the indices whose corresponding coherent states’ *X* value is samller than *P* value. We define the set $\Omega_{k}^{1}$ that includes all the indices whose corresponding coherent state’s $\Delta^{C}$ is $\leq \Delta_{k}^{C}$, and define the set $\Omega_{k}^{2}$ that includes all the indices whose corresponding coherent state’s $\Delta^{C}$ is $\geq \Delta_{k}^{C}$.

First, we try to calculate the mutual information between Alice and Bob for DMCS-BE-QKD with an arbitrary modulation. We can easily write down all the conditional probabilities of Bob’s meansurement results when using the homodyne detection and ignoring the detection efficiency and the electrical noise,

$$\begin{array}{c} \begin{array}{l} p\left(\beta_{y}=m|\alpha_{k},\;0_{B}\right)=N_{\mathrm{pdf}}\left(m,\;2\sqrt{T}ℜ\left(\alpha_{k}\right),\;1+\mathrm{T\varepsilon}\right)\\ p\left(\beta_{y}=m|\alpha_{k},\;1_{B}\right)=N_{\mathrm{pdf}}\left(m,\;2\sqrt{T}ℑ\left(\alpha_{k}\right),\;1+\mathrm{T\varepsilon}\right), \end{array} \end{array}$$ (25)

Then, we can calculate the probabilities of the subchannel occurrence and probabilities of Bob encoding the key “0” and “1” under the subchannel,

$$\begin{array}{c} p\left(\beta_{y}=m\right)=\sum_{k,n}p\left(\alpha_{k},\;n_{B}\right)p\left(\beta_{y}=m|\alpha_{k},\;n_{B}\right) \end{array}$$ (26)

$$\begin{array}{c} p\left(0_{B}|\beta_{y}=m\right)=\frac{\sum_{k}p\left(\alpha_{k},\;0_{B}\right)p\left(\beta_{y}=m|\alpha_{k},\;0_{B}\right)}{p\left(\beta_{y}=m\right)} \end{array}$$ (27)

$$\begin{array}{c} p\left(1_{B}|\beta_{y}=m\right)=\frac{\sum_{k}p\left(\alpha_{k},\;1_{B}\right)p\left(\beta_{y}=m|\alpha_{k},\;1_{B}\right)}{p\left(\beta_{y}=m\right)} \end{array}$$ (28)

We try to calculate the conditional error rate $p\left(1_{A}|0_{B},\;\beta_{y}=m\right)$ and $p\left(0_{A}|1_{B},\;\beta_{y}=m\right)$. We will divide the situation into $M_{1}+1$ types, namely, $m<2\sqrt{T}\Delta_{0}^{C}$, $2\sqrt{T}\Delta_{0}^{C}<m<2\sqrt{T}\Delta_{1}^{C}$, $2\sqrt{T}\Delta_{1}^{C}<m<2\sqrt{T}\Delta_{2}^{C}$,..., $2\sqrt{T}\Delta_{M_{1}-2}^{C}<m<2\sqrt{T}\Delta_{M_{1}-1}^{C}$, $m>2\sqrt{T}\Delta_{M_{1}-1}^{C}$.

When $m<2\sqrt{T}\Delta_{0}^{C}$, we can obtain that,

$$\begin{array}{c} p\left(1_{A}|0_{B},\;\beta_{y}=m\right)=\frac{\sum_{k\in K_{x>p}}p\left(\alpha_{k},\;0_{B}\right)p\left(\beta_{y}=m|\alpha_{k},\;0_{B}\right)}{p\left(\beta_{y}=m\right)p\left(0_{B}|\beta_{y}=m\right)} \end{array}$$ (29)

$$\begin{array}{c} p\left(0_{A}|1_{B},\;\beta_{y}=m\right)=\frac{\sum_{k\in K_{x<p}}p\left(\alpha_{k},\;1_{B}\right)p\left(\beta_{y}=m|\alpha_{k},\;1_{B}\right)}{p\left(\beta_{y}=m\right)p\left(1_{B}|\beta_{y}=m\right)} \end{array}$$ (30)

When $2\sqrt{T}\Delta_{k}^{C}<m<2\sqrt{T}\Delta_{k+1}^{C}$ ($0\leq k\leq M_{1}-2$), we can obtain that,

$$\begin{array}{c} \begin{array}{c} p\left(1_{A}|0_{B},\;\beta_{y}=m\right)=\frac{\sum_{k\in \left(\Omega_{k+1}^{2}\cap K_{x>p}\right)\cup \left(\Omega_{k}^{1}\cap K_{x<p}\right)}p\left(\alpha_{k},\;0_{B}\right)p\left(\beta_{y}=m|\alpha_{k},\;0_{B}\right)}{p\left(\beta_{y}=m\right)p\left(0_{B}|\beta_{y}=m\right)} \end{array} \end{array}$$ (31)

$$\begin{array}{c} \begin{array}{c} p\left(0_{A}|1_{B},\;\beta_{y}=m\right)=\frac{\sum_{k\in \left(\Omega_{k+1}^{2}\cap K_{x<p}\right)\cup \left(\Omega_{k}^{1}\cap K_{x>p}\right)}p\left(\alpha_{k},\;1_{B}\right)p\left(\beta_{y}=m|\alpha_{k},\;1_{B}\right)}{p\left(\beta_{y}=m\right)p\left(1_{B}|\beta_{y}=m\right)} \end{array} \end{array}$$ (32)

When $m>2\sqrt{T}\Delta_{M_{1}-1}^{C}$, we can obtain that,

$$\begin{array}{c} p\left(1_{A}|0_{B},\;\beta_{y}=m\right)=\frac{\sum_{k\in K_{x<p}}p\left(\alpha_{k},\;0_{B}\right)p\left(\beta_{y}=m|\alpha_{k},\;0_{B}\right)}{p\left(\beta_{y}=m\right)p\left(0_{B}|\beta_{y}=m\right)} \end{array}$$ (33)

$$\begin{array}{c} p\left(0_{A}|1_{B},\;\beta_{y}=m\right)=\frac{\sum_{k\in K_{x>p}}p\left(\alpha_{k},\;1_{B}\right)p\left(\beta_{y}=m|\alpha_{k},\;1_{B}\right)}{p\left(\beta_{y}=m\right)p\left(1_{B}|\beta_{y}=m\right)} \end{array}$$ (34)

and thus, we can obtain that,

$$\begin{array}{c} p\left(0_{A}|\beta_{y}=m\right)=\sum_{k}p\left(k_{B}|\beta_{y}=m\right)p\left(0_{A}|k_{B},\;\beta_{y}=m\right) \end{array}$$ (35)

$$\begin{array}{c} p\left(1_{A}|\beta_{y}=m\right)=\sum_{k}p\left(k_{B}|\beta_{y}=m\right)p\left(1_{A}|k_{B},\;\beta_{y}=m\right) \end{array}$$ (36)

Above all, the mutual information between Alice and Bob for DMCS-BE-QKD with an arbitrary modulation is given by,

$$\begin{array}{c} I\left(A;\;B|\beta_{y}=m\right)=H\left(A|\beta_{y}=m\right)-H\left(A|B,\;\beta_{y}=m\right) \end{array}$$ (37)

When keeping the modulation constellation unchanged, just changing the detecting method as heterodyne detecting or considering the detection efficiency and the electrical noise, we can just obey the rule discussed in Supplementary Note I to replace *T* and ε in Eqs. 25 to 37.

We can use the completely same process discussed above to calculate the leaked information to Eve under individual and collective attacks with an arbitrary modulation. Finally, we are able to calculate the secret key rate in this situation according to Eqs. 23 and 24.

### The simulation performance

**Fig. 4.**
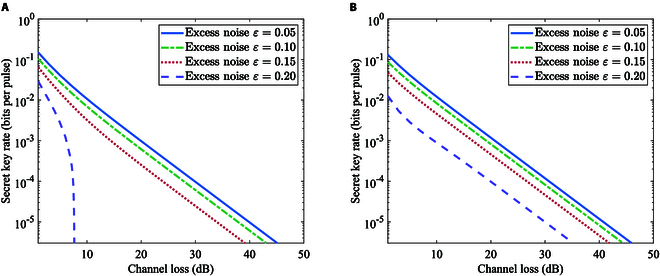
Secret key rates of (A) BPSK-BE-QKD or (B) QPSK-BE-QKD via the channel loss for different channel excess noises in the linear Gaussian channel under individual attacks. The modulation variance is (A) $V_{A}$ = 0.5 or (B) $V_{A}$ = 1.

In this section, we perform the numerical simulation of secret key rates for the B/QPSK-BE-QKD protocol in the linear Gaussian channel under individual and collective attacks by the method described above. We calculate secret key rates for the original B/QPSK-CVQKD in the linear channel under collective attacks according to [6]. Since we mainly focus on the physical characteristics of the proposed protocol, the reconciliation efficiency is set to 1 for both the BE scheme and the original scheme in the following simulations. It is noted that the photon number cutoff and the finite number of subchannels have an impact on the security. We observe that the secret key rate does not depend on the specific value of the photon number cutoff parameter *N* and the number of subchannels provided that they are both larger than 8. Figure 4 shows the secret key rates of B/QPSK-BE-QKD via the channel loss for different channel excess noises in the linear Gaussian channel under individual attacks. Both BPSK and QPSK BE-QKD protocols show high performance in the noise tolerance and the transmission distance. Meanwhile, QPSK-BE-QKD performs better than the BPSK-BE-QKD under individual attacks.

**Fig. 5.**
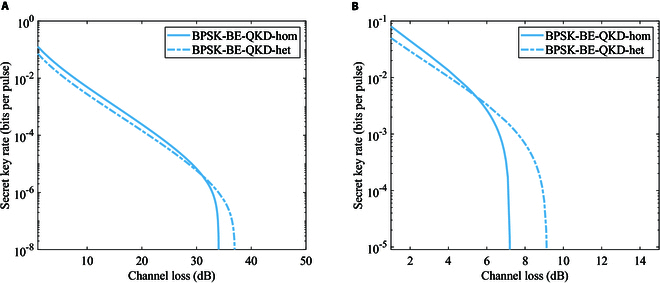
Secret key rates of BPSK-BE-QKD via the channel loss for the excess noise (A) *ε* =0.02 or (B) *ε* = 0.05 in the linear Gaussian channel under collective attacks. Here, we ignore the detection efficiency and the electrical noise. The modulation variance is $V_{A}$ = 0.5 for BPSK-BE-QKD.

As depicted in Fig. 5, the BPSK-BE-QKD both for homodyne and heterodyne detections can withstand the approximately 30- and 7-dB channel loss under collective attacks when the excess noises ε are 0.02 and 0.05, respectively. However, when the excess noise ε is 0.02 or 0.05, the original BPSK-CVQKD cannot code even when the channel loss is 0 dB. Meanwhile, Fig. S4 shows similar results when considering the effect of the detection efficiency and the electrical noise. Key rate curve for BPSK-BE-QKD with more excess noise selections is shown in Supplementary Note VI. It can be seen that BPSK-BE-QKD is less sensitive to the excess noise compared to the original BPSK-CVQKD.

**Fig. 6.**
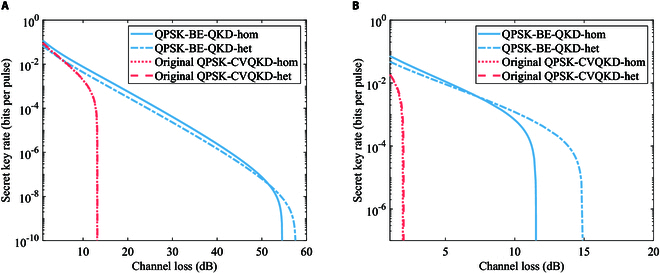
Secret key rates of QPSK-BE-QKD via the channel loss for the excess noise (A) *ε* = 0.02 or (B) *ε* = 0.05 in the linear Gaussian channel under collective attacks. Here, we ignore the detection efficiency and the electrical noise. The modulation variance is $V_{A}$ = 1 for QPSK-BE-QKD. For original QPSK-CVQKD, we set its modulation variance $V_{A}$ to the optimal value obtained through traversal.

As shown in Fig. 6, QPSK-BE-QKD can withstand the approximately 50- and 10-dB channel loss under collective attacks when the excess noise ε is 0.02 and 0.05, respectively. QPSK-BE-QKD can tolerate approximately 40 dB and 10 more channel loss compared to the original QPSK-CVQKD when excess noises ε are 0.02 and 0.05, respectively (for standard optical fibers, it is equivalent to extending the distance for about 200 and 50 km, respectively). It shows similar results when considering the imperfection of the detection according to Fig. S5. Key rate curve for QPSK-BE-QKD with more excess noise selections is shown in Supplementary Note VI. It is noted that the low bound of the key rate obtained in [6] is not tight enough, and both the BE protocol itself and the security analysis method may contribute to these observed improvements in terms of the tolerable channel loss and excess noise. The exact proportion of the contribution that the above 2 factors make is still unknown and requires further study. In addition, it is noted that Lin et al. [17] reflected the best performance of the current QPSK-CVQKD scheme under the general collective attack. According to the above results, QPSK-BE-QKD with linear Gaussian channel assumption can tolerate several dB more channel loss compared to the result in [17]. We will broadly discuss the performance potential of QPSK-BE-QKD if they are all under general collective attack conditions. Under the general collective attack, we need to traverse all possible states at ${\mathrm{AB}}_{1}$ and all possible purification of $\rho_{{\mathrm{AB}}_{1}}$ (since the state at Eve can be any purification of $\rho_{{\mathrm{AB}}_{1}}$), and then the secret key rate is calculated using the maximum corresponding leaked information. We prove that the leaked information for DMCS-BE-QKD remains unchanged for different purification of $\rho_{{\mathrm{AB}}_{1}}$ in Supplementary Note V and conduct the partly traverse for possible $\rho_{{\mathrm{AB}}_{1}}$ by the semi-definite programming [17]. As shown in Fig. S6, it can be seen that all the secret key rates for QPSK-BE-QKD under possible collective attacks we partly traversed are not less than the secret key rate under the collective entangling clone attack. This suggests that QPSK-BE-QKD has some potential to have a performance advantage over conventional QPSK-CVQKD under general collective attacks. It can also be seen from Fig. 6 that QPSK-BE-QKD can achieve the higher secret key rate and the longer distance than the BPSK-BE-QKD under collective attacks, which is consistent with the conclusion obtained under individual attacks. This is because the overall mutual information between Alice and Bob for QPSK-BE-QKD and BPSK-BE-QKD can be considered approximately equal (because the overall average bit error rates between Alice and Bob for these 2 protocols are completely the same, where the bit error rate only depends on the absolute value of the difference between the *X* and *P* components of each constellation point). At the same time, the constellation diagram of QPSK-BE-QKD will decrease the orthogonality between the conditional quantum states $\rho_{E}^{x=m}$ and $\rho_{E}^{p=m}$ while making it more difficult for Eve to conduct the maximum mutual information discrimination of $\rho_{E}^{x=m}$ and $\rho_{E}^{p=m}$. Above all, B/QPSK-BE-QKD can quite improve the secure transmission distance and the tolerable channel excess noise.

**Fig. 7.**
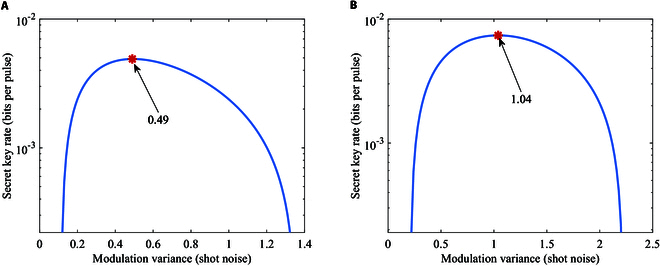
Secret key rates of (A) BPSK-BE-QKD or (B) QPSK-BE-QKD via the modulation variance with homodyne detection in the linear Gaussian channel under collective attacks. The channel loss is *T* = 10 dB, and the excess noise is *ε* = 0.02.

Figure 7 shows secret key rates of B/QPSK-BE-QKD via the modulation variance in the linear Gaussian channel under collective attacks. The result shows that the modulation variance $V_{A}$ has its best value for B/QPSK-BE-QKD. The best value of the modulation variance $V_{A}$ is 0.49 and 1.04 for BPSK-BE-QKD and QPSK-BE-QKD, respectively.

**Fig. 8.**
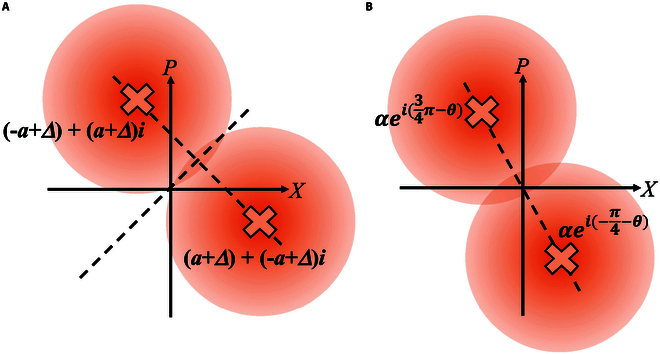
Illustration of (A) the translation of constellation points and (B) the rotation of constellation points for BPSK-BE-QKD.

**Fig. 9.**
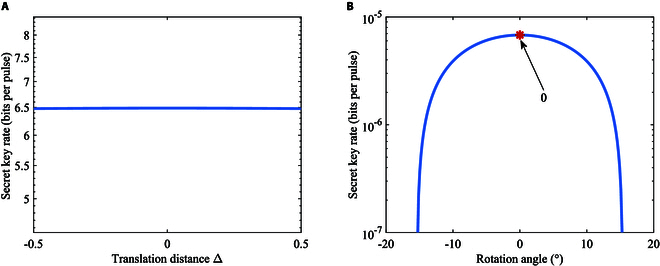
Secret key rates of BPSK-BE-QKD with homodyne detection via (A) the translation distance Δ or (B) the rotation angle in the linear Gaussian channel under collective attacks. The channel loss is *T* = 30 dB, the excess noise is *ε* = 0.02, and the modulation variance $V_{A}$ = 0.5.

Then, we further study the relationship between the modulation constellation and the secret key for BPSK-BE-QKD. As shown in Fig. 8A, we translate the constellation points along the y=x axis. The calculation of the mutual information between Alice and Bob in this situation is detailed in Supplementary Note I. The calculation of the leaked information to Eve for this situation is completely the same with the discussion above, because changing the constellation diagram only changes $\alpha_{k}$ in Eq. 12, and we can still use Eqs. 3, 12, to 22 to calculate the leaked information. Figure 9A shows secret key rates of BPSK-BE-QKD via the translation distance Δ. It can be seen that translating the constellation points along the y=x axis will not influence the secret key rate for BPSK-BE-QKD. Because no matter how the constellation points are translated along the y=x axis, the probability density curves of the corresponding detection values when Bob performs the *X* and *P* measurements are always exactly the same. So, translation along the y=x axis will not affect the performance of judging whether Bob performs an *X* or *P* measurement for both Alice and Eve and thus will not affect the secret key rate. Then, we rotate the constellation points around the original point as depicted in Fig. 8B. The calculation of the mutual information between Alice and Bob for this constellation is detailed in Supplementary Note I. As shown in Fig. 9B, the more rotations relative to the y=−x axis, the lower the secret key rate is. When constellation points are located at y=−x, i.e., the rotation angle is 0, the secret key rate achieves its maximum. We can find that the symmetry relative to the y=x axis is beneficial for BPSK-BE-QKD. Because as the rotation angle increases, the difference between the probability density curves of the corresponding detection values when Bob performs the *X* and *P* measurements will become larger. This will help Eve gain an information advantage in the base selection information, resulting in a relatively lower secret key rate.

**Fig. 10.**
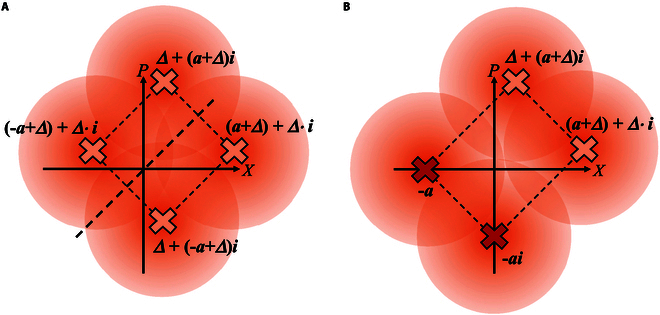
Illustration of (A) translating all the constellation points while fixing the constellation diagram as a regular prism and (B) translating 2 of the constellation points while fixing the other 2 constellation points at (−a, *0*) and (0, −ai) for QPSK-BE-QKD.

**Fig. 11.**
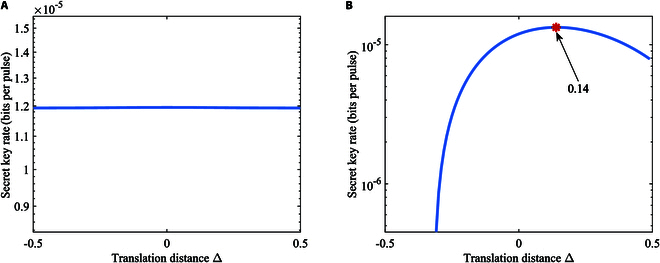
Secret key rates of QPSK-BE-QKD with homodyne detection via the translation distance in the linear Gaussian channel under collective attacks when (A) translating all the constellation points while fixing the constellation diagram as a regular prism or (B) translating 2 of the constellation points while fixing the other 2 constellation points at (−a, *0*) and (0, −ai). The channel loss is *T* = 30 dB, the excess noise is *ε* = 0.02, and the modulation variance $V_{A}$ = 0.5.

We finally study the relationship between the modulation constellation and the secret key for QPSK-BE-QKD. We translate the constellation points in 2 different ways. As depicted in Fig. 10A, in the first way, we translate all the constellation points along the y=x axis while fixing the constellation as a regular prism. In the second way, we translate 2 of the constellation points along the y=x axis while fixing the other 2 constellation points at (−a, *0*) and (0, −ai), as shown in Fig. 10B. As depicted in Fig. 11A, we observe that translating all the constellation points along the y=x axis does not affect the secret key rate, similar to the protocol property observed in BPSK-BE-QKD. It is because of the similar reason discussed for Fig. 9A, i.e., the probability density curves of the corresponding detection values when Bob performs the *X* and *P* measurements are always exactly the same while translating all the constellation points along the y=x axis. As shown in Fig. 11B, we know that if we fix 2 constellation points and translate the other 2 constellations along the y=x axis, the secret key rate has its maximum value and the translation distance has its best value (i.e., the modulation constellation has its best shape). We can find that in this situation, the best shape of the modulation constellation is the rectangle. This is because only translating 2 of the constellation diagram’s 4 points along the y=x axis will cause the amount of information leaked to Eve to first decrease and then increase. Meanwhile, the overall mutual information between Alice and Bob can be seen as approximately equal, because the overall average bit error rate between Alice and Bob remains completely unchanged. Thus, the secret key rate will first increase and then decrease.

### Experimental demonstration

**Fig. 12.**
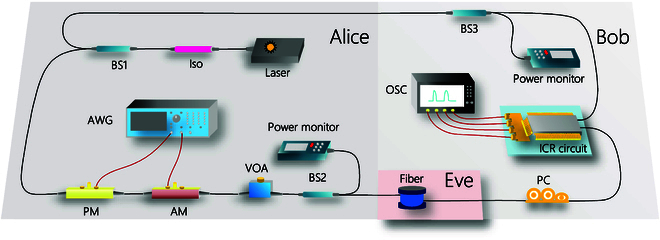
Experimenntal demonstration of QPSK-BE-QKD.

To verify the feasibility of the BE-QKD protocol, we conduct an experiment demonstration of QPSK-BE-QKD under the 50.5-km optical fiber. The experiment step of QPSK-BE-QKD is demonstrated in Fig. 12. At the Alice site, a narrow-linewidth continous-wave (CW) laser with 100-Hz linewidth (NKT Koheras BASIK X15) is used to generate the optical carrier, followed by an optical isolator (ISO) to prevent the reflected light transmitting into the laser. Then, the optical light is splitted into 2 parts by the 50:50 beam splitter 1 (BS1). One part is transmitted to the Bob site to serve as a local osillator (LO), and the 90:10 BS3 is deployed to monitor the power of the LO. The other part served as the signal (SIG). Here, we used the pilot sequence scheme [48], where we alternately generate the pilot signal and the quantum signal. The amplitude of the pilot signal is 10 times larger than the quantum signal. It is used as the reference signal with the fixed amplitude and phase to help find the peak point of the pulse for Bob and correct the fast phase drift of quantum signals [48] due to the inconsistency in the arrival time of LO and SIG light at the receiver. SIG light is first sent into the phase modulator (PM; EOSPACE) to achieve the QPSK modulation (modulated as 4 states $|a\rangle$, $|\mathrm{ai}\rangle$, $|\;-\;a\rangle$, and $|\;-\;\mathrm{ai}\rangle$, where $a=\sqrt{V_{A}/2}$). Meanwhile, an arbitrary wave generator (AWG; Tektronix, AWG5200) is used to output a electronic analog signals $\theta \left(t\right)$ with the 50-MHz symbol rate to the PM, where $\theta \left(t\right)$ is controlled by random number data. When the random number is $\left\{0,\;1,\;2,\;3\right\}$, $\theta \left(t\right)=\left\{0,\frac{V_{\pi }}{2},V_{\pi },-\frac{V_{\pi }}{2}\right\}$, where $V_{\pi }$ is the half-wave voltage for PM. An ultra-high-extinction-ratio amplitude modulator (AM; EOSPACE) is deployed to cut the CW SIG light into pulse light with the 20% duty cycle. It is also controlled by the electronic analogy signal generated by the AWG, and its direct current (DC) bias voltage is provided by a DC stabilized power. Moreover, the phase of the modulation of the pilot signal is fixed to 0 (i.e., $\theta \left(t\right)=0$), and we make its amplitude 10 times that of the quantum signal by the AM. Then, the SIG light is attenuated by variable optical attenuator (VOA) to control the modulation variance $V_{A}$. A 90:10 BS2 monitors the channel input optical power in real time, followed by a 50.5-km fiber channel with 11 dB. It is noted that the channel is assumed to be fully controlled by Eve.

At the Bob site, a polarization controller (PC) is deployed to adjust the polarization of the SIG light to minimize the component on the *V* polarization of the SIG light as much as possible. Then, the LO and SIG lights are injected into the integrated conherent receiver (ICR; FUJITSU) circuit to measure the *X* and *P* quadratures, and randomly choose one of them as final measurement results. The detected signals are sampled by a real-time oscilloscope (OSC; Lecroy, WaveRunner 9404M-MS) with 1 GSamples/s sampling rate (20 sample points for one symbol). In the end, Bob announces the measurement results publicly and Alice tries to guess the measurement bases according to the decoding rule. The quantum efficiency is η=0.48. The electrical noise $v_{\mathrm{el}}=0.0997$ (shot noise units), which is calibrated by sampling data without connecting the LO. Moreover, the frame length we used is10^5^.

Then, we first need to find the peak point for each pulse. We calculate the power of the first 4 × 10^5^ sampling data and find its maximum value $P_{i}$. The corresponding index *i mod 20* is the optimal sampling position. Then, we can extract the final sampling data at every 20 points from the optimal sampling position. Then, we use the pilot signal data to correct the fast phase drift of the quantum signal data. We directly use the phase angle of the 2 pilot signals to rotate the quantum signal and recover the quantum signal with the following formula:

$$\begin{array}{c} A_{j}^{\mathrm{rot}}=\sqrt{\frac{x_{j}^{p}+{\mathrm{ip}}_{j}^{p}}{\left|x_{j}^{p}+{\mathrm{ip}}_{j}^{p}\right|}}\cdot \sqrt{\frac{x_{j+1}^{p}+{\mathrm{ip}}_{j+1}^{p}}{\left|x_{j+1}^{p}+{\mathrm{ip}}_{j+1}^{p}\right|}} \end{array}$$ (38)

$$x_{j}^{q}+{\mathrm{ip}}_{j}^{q}\leftarrow \frac{x_{j}^{q}+{\mathrm{ip}}_{j}^{q}}{A_{j}^{\mathrm{rot}}}$$ (39)

where $x_{j}^{p}$ and $p_{j}^{p}$ are the *X* and *P* component of the *j*th pilot signal, and $x_{j}^{q}$ and $p_{j}^{q}$ are the *X* and *P* components of the *j*th quantum signal.

**Fig. 13.**
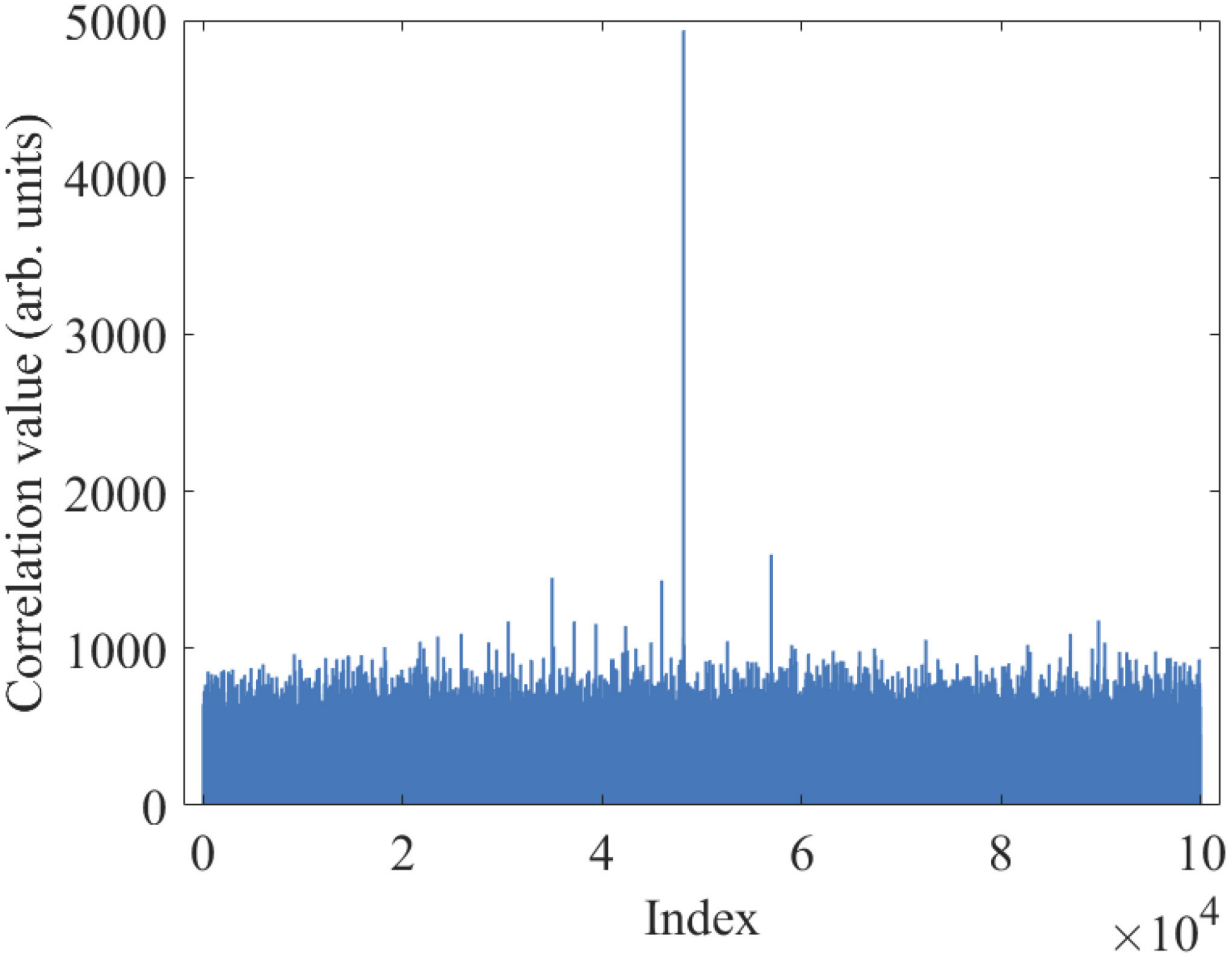
Correlation value of the frame synchronization sequence and the received frame at different indices.

**Fig. 14.**
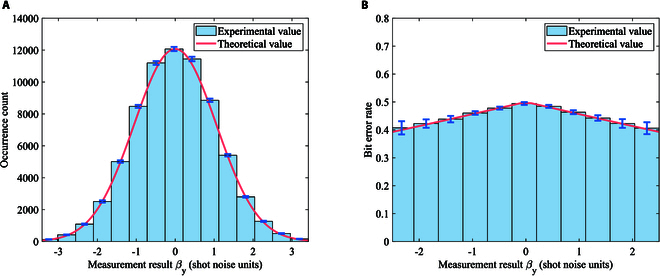
The experimental and theoretical value of (A) the occurrence count and (B) the bit error rate via different measurement results $\beta_{y}$. The error bars denote the standard deviation (SD) of the experimental data.

**Fig. 15.**
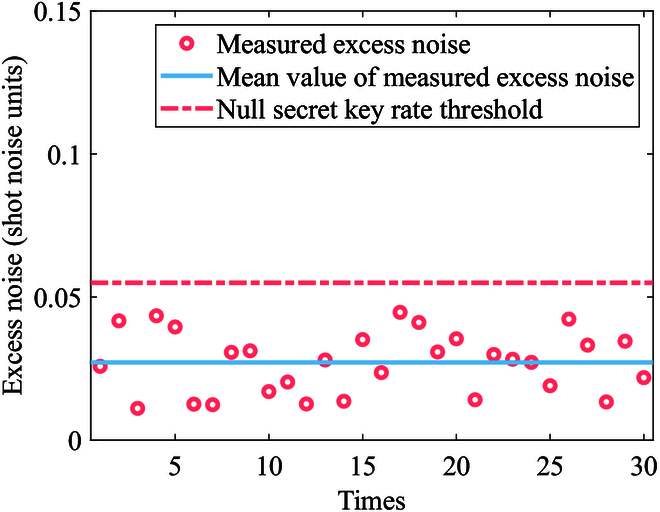
Measured excess noise. The red circles represent the measured excess noise. The blue solid line represents the mean value of the measured excess noise. The red dash-dot line represents the excess noise threshold of the null secret key rate.

**Fig. 16.**
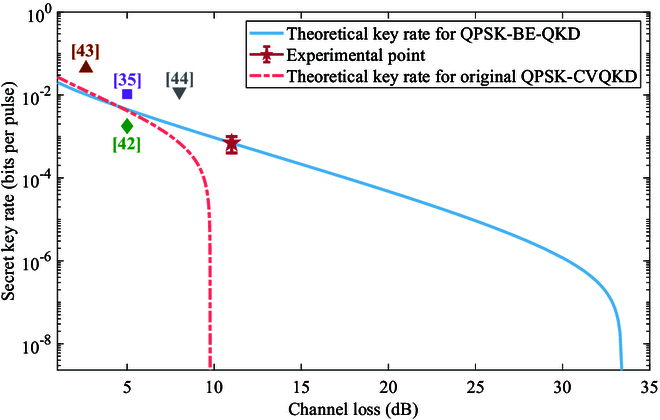
Secret key rate via the channel loss. The red star point corresponds to the experimental result under the 50.5-km optical fiber. The blue solid curve shows the simulated secret key rates of QPSK-BE-QKD calculated from estimated parameters in the experiment. The red dash-dot curve shows the simulated secret key rates of the original QPSK-CVQKD calculated from the same parameters estimated in the experiment except the modulation variance $V_{A}$ (we set its $V_{A}$ to the optimal value obtained through traversal). Purple squares, green prisms, brown upper triangles, and gray lower triangles represent results in [35,42–44], respectively. The error bars denote the SD of the experimental data.

In order to obtain the correlation between modulation data and receive data, we need to achieve the precise frame synchronization. We use a robust synchronizaion method proposed in [49] to find the frame header. The synchronization result is shown in Fig. 13. We observe that the correlation between the frame synchronization sequence and the received frame reaches its peak at index 48159, which is significantly higher than other indices. So, we successfully achieve the frame synchronization to get the correlation between modulation data and receive data. Then, we compensate the slow phase drift due to the slow fluctuation of the transmission path length [50]. We also analyze the occurrence probabilities and the bit error rate between Alice and Bob for different measurement results $\beta_{y}$, as depicted in Fig. 14. The results show that the experimental value is consistent with the theoretical curve, where the theortical curve can be calculated according to the mutual information analysis above. Finally, we conduct the parameter evaluation, and the measured excess noises of 30 frames are shown in Fig. 15. We get that the mean modulation variance $V_{A}$ is 1.003 and the mean excess noise ε is 0.02716. Meanwhile, we use a high-efficient information reconcilization algorithm [51] to make the reconcilization efficiency β reach 96.58%. Based on the calculation method for the secret key rate discussed above, we finally obtain the 13.12-kbps secret key rate under the 50.5-km optical fiber with 11-dB loss, as shown in Fig. 16. Based on the estimated parameters in the experiment, our QPSK-BE-QKD can theoretically sustain secure key generation under the 33-dB channel loss. It is noted that based on the same parameters estimated in our experiment except the modulation variance $V_{A}$ (we set its $V_{A}$ to the optimal value obtained through traversal), the original QPSK-CVQKD can only tolerate a maximum channel loss of approximately 10 dB.

## Discussion

We have provided a security analysis of DMCS-BE-QKD protocol under individual and collective attacks in the linear Gaussian channel. The simulation result shows that the B/QPSK-BE-QKD protocol shows the better tolerance for the channel loss and excess noise than the original B/QPSK-CVQKD. The result shows that the B/QPSK-BE-QKD has potential to surpass the original B/QPSK-CVQKD without the channel assumption. We also conduct a proof-of-principle experiment for QPSK-BE-QKD under a 50.5-km optical fiber with 11-dB loss to prove the feasibility of the BE scheme. Since the quantum communication part of the BE-QKD scheme is the same as the traditional CVQKD, one can naturally choose the basis encoding or the traditional way in the practical application to optimize the secret key rate after the parameter estimation. Thus, the BE-QKD scheme can be perfectly compatible with the existing CVQKD terminals and applied in quantum access networks and quantum metropolitan networks, further promoting the quantum Internet as a reality. In addition, the BE scheme, as well as the security analysis framework proposed in this paper, have the potential to be applied to other QKD protocols and quantum secure direct communication [52–54] to further improve their performance. In conclusion, we believe the BE-QKD scheme provides an efficient way for practical secure quantum cryptography communication in large-scale and harsh environments.

In the future, we will try to analyze the secret key rate of DMCS-BE-QKD under collective attacks without the channel assumption. Theoretically, we need to traverse all possible states at ${\mathrm{AB}}_{1}$ and all possible purifications of $\rho_{{\mathrm{AB}}_{1}}$, and then use the one that will maximize the leaked information to Eve to calculate the leaked information. In Supplementary Note V, we prove that the leaked information remains unchanged for different purifications of $\rho_{{\mathrm{AB}}_{1}}$ under collective attacks. So, we just need to traverse all possible states at ${\mathrm{AB}}_{1}$. We obtain hundreds of feasible $\rho_{{\mathrm{AB}}_{1}}$ under the constraints [17] for the general channel by the semi-definite programming and calculate the corresponding secret key rates. The simulation result shows that secret key rates under possible collective attacks we partly traversed are not less than the secret key rate under the collective entangling clone attack. This partly indicates that the collective entangling clone attack is likely to be the optimal attack. To traverse all possible states at ${\mathrm{AB}}_{1}$, we will try more related numerical methods to solve this problem.

## Data Availability

All of the data that support the findings of this study are reported in the main text and the Supplementary Materials. Source data are available from the corresponding authors on reasonable request.

## Supplementary Materials

Supplementary 1 Supplementary Notes I to VIII Figs. S1 to S6
