# Supplementary material for: Discrete-Modulated Coherent-State Quantum Key Distribution with Basis-Encoding
Source: Research (Wash D C). 2025 May 14;8:0691. doi: 10.34133/research.0691 (PMC12076462; doi:10.34133/research.0691)
Supplement: Supplementary 1 — Supplementary Notes I to VIII Figs. S1 to S6 [file research.0691.f1.pdf]

Supplemental Material for Discrete-Modulated Coherent-State Quantum Key Distribution With Basis-Encoding

Mingxuan Guo[1], Peng Huang[1,2,3*], Le Huang[1], Xiaojuan Liao[1], Xueqin Jiang[4,2,3], Tao Wang[1,2,3], Guihua Zeng[1,2,3*]

[1]State Key Laboratory of Photonics and Communications, Shanghai Jiao Tong University, Shanghai, 200240, China

[2]Shanghai Research Center for Quantum Science, Shanghai, 201315, China

[3]Hefei National Laboratory, Hefei, 230088, China

[4]College of Information Science and Technology, Donghua University, Shanghai, 201620, China

huang.peng@sjtu.edu.cn; ghzeng@sjtu.edu.cn;

Supplementary Note I: Mutual information between Alice and Bob

The mutual information between Alice and Bob for BPSK-BE-QKD is discussed firstly. Here, the simplest situation is discussed, i.e., the modulation constellation ($\alpha_0 = -a + ai$, $\alpha_1 = a - ai$) shown as Fig. 3(a) in the main text is used, Bob uses the homodyne detection, and the detection efficiency and the electrical noise are ignored. Firstly, we can give out the conditional probabilities of Bob's measurement results,

$$
\begin{aligned}
&p(\beta_y = m|\alpha_0, 0_B) = N_{pdf}(m, -2\sqrt{T}a, 1 + T\varepsilon) = p_1,\\
&p(\beta_y = m|\alpha_1, 0_B) = N_{pdf}(m, 2\sqrt{T}a, 1 + T\varepsilon) = p_2,\\
&p(\beta_y = m|\alpha_0, 1_B) = N_{pdf}(m, 2\sqrt{T}a, 1 + T\varepsilon) = p_2,\\
&p(\beta_y = m|\alpha_1, 1_B) = N_{pdf}(m, -2\sqrt{T}a, 1 + T\varepsilon) = p_1,
\end{aligned}
\tag{1}
$$

We can then write down the probability of the sub-channel occurrence,

$$
p(\beta_y = m) = \sum_{k,n} p(\alpha_k, n_B) p(\beta_y = m|\alpha_k, n_B) = 0.5(p_1 + p_2). \tag{2}
$$

Then, we can calculate the probabilities of encoding "0_B" and "1_B" under the sub-channel,

$$
p(0_B|\beta_y = m) = \frac{\sum_k p(\alpha_k, 0_B) p(\beta_y = m|\alpha_k, 0_B)}{p(\beta_y = m)} = \frac{0.25(p_1 + p_2)}{0.5(p_1 + p_2)} = 0.5, \tag{3}
$$

$$
p(1_B|\beta_y = m) = \frac{\sum_k p(\alpha_k, 1_B) p(\beta_y = m|\alpha_k, 1_B)}{p(\beta_y = m)} = \frac{0.25(p_1 + p_2)}{0.5(p_1 + p_2)} = 0.5. \tag{4}
$$

In order to calculate the mutual information between Alice and Bob, then we need to calculate the conditional error rate $p(1_A|0_B, \beta_y = m)$ when Bob encodes the key "0" and $p(0_A|1_B, \beta_y = m)$ when Bob encodes the key "1". We will divide the situation into two types, namely $m > 0$ and $m < 0$.

When $m > 0$, we can obtain that,

$$
p(1_A|0_B, \beta_y = m) = \frac{p(\alpha_0, 0_B) p(\beta_y = m|\alpha_0, 0_B)}{p(\beta_y = m) p(0_B|\beta_y = m)} = \frac{0.25 p_1}{0.25(p_1 + p_2)} = \frac{1}{1 + e^{\frac{4\sqrt{T}am}{1+T\varepsilon}}} = \frac{1}{1 + e^{|\frac{4\sqrt{T}am}{1+T\varepsilon}|}}, \tag{5}
$$

$$
p(0_A|1_B, \beta_y = m) = \frac{p(\alpha_1, 1_B) p(\beta_y = m|\alpha_1, 1_B)}{p(\beta_y = m) p(1_B|\beta_y = m)} = \frac{0.25 p_1}{0.25(p_1 + p_2)} = \frac{1}{1 + e^{\frac{4\sqrt{T}am}{1+T\varepsilon}}} = \frac{1}{1 + e^{|\frac{4\sqrt{T}am}{1+T\varepsilon}|}}. \tag{6}
$$

When $m < 0$, we can obtain that,

$$
p(1_A|0_B, \beta_y = m) = \frac{p(\alpha_1, 0_B) p(\beta_y = m|\alpha_1, 0_B)}{p(\beta_y = m) p(0_B|\beta_y = m)} = \frac{0.25 p_2}{0.25(p_1 + p_2)} = \frac{1}{1 + e^{-\frac{4\sqrt{T}am}{1+T\varepsilon}}} = \frac{1}{1 + e^{|\frac{4\sqrt{T}am}{1+T\varepsilon}|}}, \tag{7}
$$

$$
p(0_A|1_B, \beta_y = m) = \frac{p(\alpha_0, 1_B) p(\beta_y = m|\alpha_0, 1_B)}{p(\beta_y = m) p(1_B|\beta_y = m)} = \frac{0.25 p_2}{0.25(p_1 + p_2)} = \frac{1}{1 + e^{-\frac{4\sqrt{T}am}{1+T\varepsilon}}} = \frac{1}{1 + e^{|\frac{4\sqrt{T}am}{1+T\varepsilon}|}}. \tag{8}
$$

We define that,

$$
p_{error}^{BPSK} = 1/(1 + \exp|\frac{4\sqrt{T}am}{1 + T\varepsilon}|), \tag{9}
$$

thus we can get that,

$$p(1_A|0_B,\beta_y=m)=p(0_A|1_B,\beta_y=m)=p_{error}^{BPSK}. \tag{10}$$

Furthermore, we can calculate that,

$$p(0_A|\beta_y=m)=\sum_k p(k_B|\beta_y=m)p(0_A|k_B,\beta_y=m)=0.5, \tag{11}$$
$$p(1_A|\beta_y=m)=\sum_k p(k_B|\beta_y=m)p(1_A|k_B,\beta_y=m)=0.5. \tag{12}$$

Above all, the mutual information between Alice and Bob for BPSK-BE-QKD is given by,

$$I(A;B|\beta_y=m)=H(A|\beta_y=m)-H(A|B,\beta_y=m)=1-H(p_{error}^{BPSK}). \tag{13}$$

Then, the mutual information between Alice and Bob for QPSK-BE-QKD is discussed. We discuss the situation that the modulation constellation ($\alpha_0=-a+\Delta a+(a+\Delta a)i$, $\alpha_1=-a+\Delta b+(a+\Delta b)i$, $\alpha_2=a+\Delta b+(-a+\Delta b)i$, $\alpha_3=a+\Delta a+(-a+\Delta a)i$, $\Delta a>\Delta b$) shown as Fig. 3(b) in the main text is used, Bob uses the homodyne detection, and the detection efficiency and the electrical noise are ignored. We can write down the conditional probabilities of Bob's measurement results,

$$\begin{aligned}
p(\beta_y=m|\alpha_0,0_B)&=N_{pdf}(m,2\sqrt{T}(-a+\Delta a),1+T\varepsilon)=p_1\\
p(\beta_y=m|\alpha_1,0_B)&=N_{pdf}(m,2\sqrt{T}(-a+\Delta b),1+T\varepsilon)=p_2\\
p(\beta_y=m|\alpha_2,0_B)&=N_{pdf}(m,2\sqrt{T}(a+\Delta b),1+T\varepsilon)=p_3\\
p(\beta_y=m|\alpha_3,0_B)&=N_{pdf}(m,2\sqrt{T}(a+\Delta a),1+T\varepsilon)=p_4\\
p(\beta_y=m|\alpha_0,1_B)&=N_{pdf}(m,2\sqrt{T}(a+\Delta a),1+T\varepsilon)=p_4\\
p(\beta_y=m|\alpha_1,1_B)&=N_{pdf}(m,2\sqrt{T}(a+\Delta b),1+T\varepsilon)=p_3\\
p(\beta_y=m|\alpha_2,1_B)&=N_{pdf}(m,2\sqrt{T}(-a+\Delta b),1+T\varepsilon)=p_2\\
p(\beta_y=m|\alpha_3,1_B)&=N_{pdf}(m,2\sqrt{T}(-a+\Delta a),1+T\varepsilon)=p_1.
\end{aligned} \tag{14}$$

Similarly, we can calculate probabilities of the sub-channel occurrence, probabilities of Bob encoding the key "0" and "1" under the sub-channel,

$$p(\beta_y=m)=\sum_{k,n} p(\alpha_k,n_B)p(\beta_y=m|\alpha_k,n_B)=0.25(p_1+p_2+p_3+p_4), \tag{15}$$
$$p(0_B|\beta_y=m)=\frac{\sum_k p(\alpha_k,0_B)p(\beta_y=m|\alpha_k,0_B)}{p(\beta_y=m)}=\frac{0.25(p_1+p_2+p_3+p_4)}{0.5(p_1+p_2+p_3+p_4)}=0.5, \tag{16}$$
$$p(1_B|\beta_y=m)=\frac{\sum_k p(\alpha_k,1_B)p(\beta_y=m|\alpha_k,1_B)}{p(\beta_y=m)}=\frac{0.25(p_1+p_2+p_3+p_4)}{0.5(p_1+p_2+p_3+p_4)}=0.5. \tag{17}$$

We try to calculate the conditional error rate $p(1_A|0_B,\beta_y=m)$ and $p(0_A|1_B,\beta_y=m)$. We will divide the situation into three types, namely $m>2\sqrt{T}\Delta a$, $2\sqrt{T}\Delta b<m<2\sqrt{T}\Delta a$, and $m<2\sqrt{T}\Delta b$.

When $m>2\sqrt{T}\Delta a$, we can obtain that,

$$p(1_A|0_B,\beta_y=m)=\frac{p(\beta_y,\alpha_0,0_B)+p(\beta_y,\alpha_1,0_B)}{p(\beta_y=m)p(0_B|\beta_y=m)}=\frac{p_1+p_2}{p_1+p_2+p_3+p_4}, \tag{18}$$
$$p(0_A|1_B,\beta_y=m)=\frac{p(\beta_y,\alpha_2,1_B)+p(\beta_y,\alpha_3,1_B)}{p(\beta_y=m)p(1_B|\beta_y=m)}=\frac{p_1+p_2}{p_1+p_2+p_3+p_4}. \tag{19}$$

When $2\sqrt{T}\Delta b<m<2\sqrt{T}\Delta a$, we can obtain that,

$$p(1_A|0_B,\beta_y=m)=\frac{p(\beta_y,\alpha_1,0_B)+p(\beta_y,\alpha_3,0_B)}{p(\beta_y=m)p(0_B|\beta_y=m)}=\frac{p_2+p_4}{p_1+p_2+p_3+p_4}, \tag{20}$$
$$p(0_A|1_B,\beta_y=m)=\frac{p(\beta_y,\alpha_0,1_B)+p(\beta_y,\alpha_2,1_B)}{p(\beta_y=m)p(1_B|\beta_y=m)}=\frac{p_2+p_4}{p_1+p_2+p_3+p_4}. \tag{21}$$

When $m < 2\sqrt{T}\Delta b$, we can obtain that,

$$p(1_A|0_B,\beta_y=m)=\frac{p(\beta_y,\alpha_2,0_B)+p(\beta_y,\alpha_3,0_B)}{p(\beta_y=m)p(0_B|\beta_y=m)}=\frac{p_3+p_4}{p_1+p_2+p_3+p_4}, \tag{22}$$

$$p(0_A|1_B,\beta_y=m)=\frac{p(\beta_y,\alpha_0,1_B)+p(\beta_y,\alpha_1,1_B)}{p(\beta_y=m)p(1_B|\beta_y=m)}=\frac{p_3+p_4}{p_1+p_2+p_3+p_4}. \tag{23}$$

We define that,

$$p^{QPSK}_{error}=\begin{cases}\dfrac{p_1+p_2}{p_1+p_2+p_3+p_4}, m>2\sqrt{T}\Delta a\\ \dfrac{p_2+p_4}{p_1+p_2+p_3+p_4}, 2\sqrt{T}\Delta b<m<2\sqrt{T}\Delta a\\ \dfrac{p_3+p_4}{p_1+p_2+p_3+p_4}, m<2\sqrt{T}\Delta b\end{cases}, \tag{24}$$

thus we can obtain that,

$$p(1_A|0_B,\beta_y=m)=p(0_A|1_B,\beta_y=m)=p^{QPSK}_{error}, \tag{25}$$

$$p(0_A|\beta_y=m)=\sum_k p(k_B|\beta_y=m)p(0_A|k_B,\beta_y=m)=0.5, \tag{26}$$

$$p(1_A|\beta_y=m)=\sum_k p(k_B|\beta_y=m)p(1_A|k_B,\beta_y=m)=0.5. \tag{27}$$

Above all, the mutual information between Alice and Bob for QPSK-BE-QKD is given by,

$$I(A;B|\beta_y=m)=H(A|\beta_y=m)-H(A|B,\beta_y=m)=1-H(p^{QPSK}_{error}). \tag{28}$$

For BPSK-BE-QKD, its secret key rate in the situation that the modulation constellation ($\alpha_0=-a+ai$, $\alpha_1=a-ai$) shown as Fig. 3(a) in the main text is used, Bob uses the homodyne detection, and the detection efficiency and the electrical noise are ignored has been discussed above. In this part, we first discuss the other three situations. All of these situations use the same modulation constellation shown in Fig. 3(a) in the main text, the only differences are that: Situation 1: using the heterodyne detection and ignoring the detection efficiency and the electrical noise; Situation 2: using the homodyne detection and considering the detection efficiency and the electrical noise; Situation 3: using the heterodyne detection and considering the detection efficiency and the electrical noise.

These three situations are very similar to what was discussed above, we just need to replace T and ε in the equation (9) and (13). For situation 1 that we use the heterodyne detection and ignore the detection efficiency and the electrical noise, we replace T with $0.5T$ and the other remains unchanged. For situation 2 that we use the homodyne detection and considering the detection efficiency and the electrical noise, we first replace ε with $\varepsilon+v_{el}/T$ and then replace T with ηT (v_{el} represents the electrical noise in shot noise and η represents the detection efficiency). For situation 3 that we use the heterodyne detection and considering the detection efficiency and the electrical noise, we first replace ε with $\varepsilon+v_{el}/T$ and then replace T with $0.5\eta T$. More specifically, taking situation 3 as an example, the mutual information between Alice and Bob in this situation is given by,

$$p^{BPSK}_{error}=1/(1+\exp|\frac{4\sqrt{0.5\eta T}am}{1+0.5\eta T\varepsilon+v_{el}}|), \tag{29}$$

$$I(A;B|\beta_y=m)=1-H(p^{BPSK}_{error}). \tag{30}$$

For QPSK-BE-QKD, we use the similar method. We just need to replace T and ε in the equation (14-28). When using the heterodyne detection and ignoring the detection efficiency and the electrical noise, we replace T with $0.5T$ and the other remains unchanged. When using the homodyne detection and considering the detection efficiency and the electrical noise, we first replace ε with $\varepsilon+v_{el}/T$ and then replace T with ηT. When using the heterodyne detection and considering the detection efficiency and the electrical noise, we first replace ε with $\varepsilon+v_{el}/T$ and then replace T with $0.5\eta T$.

For BPSK-BE-QKD, when using the modulation constellation ($\alpha_0=-a+\Delta a+(a+\Delta a)i$, $\alpha_1=a+\Delta a+(-a+\Delta a)i$) shown in Fig. 8(a) in the main text, the homodyne detection, and ignoring the detection efficiency and the electrical noise, we can calculate the mutual information between Alice and Bob through following formulas. Firstly, we can give out the

conditional probabilities of Bob's measurement results,

$$\begin{aligned}
&p(\beta_y=m|\alpha_0,0_B)=N_{pdf}(m,2\sqrt{T}(-a+\Delta a),1+T\varepsilon)=p_1,\\
&p(\beta_y=m|\alpha_1,0_B)=N_{pdf}(m,2\sqrt{T}(a+\Delta a),1+T\varepsilon)=p_2,\\
&p(\beta_y=m|\alpha_0,1_B)=N_{pdf}(m,2\sqrt{T}(a+\Delta a),1+T\varepsilon)=p_2,\\
&p(\beta_y=m|\alpha_1,1_B)=N_{pdf}(m,2\sqrt{T}(-a+\Delta a),1+T\varepsilon)=p_1.
\end{aligned}\tag{31}$$

We can then calculate that,

$$p(\beta_y=m)=\sum_{k,n}p(\alpha_k,n_B)p(\beta_y=m|\alpha_k,n_B)=0.5(p_1+p_2).\tag{32}$$

$$p(0_B|\beta_y=m)=\frac{\sum_k p(\alpha_k,0_B)p(\beta_y=m|\alpha_k,0_B)}{p(\beta_y=m)}=\frac{0.25(p_1+p_2)}{0.5(p_1+p_2)}=0.5,\tag{33}$$

$$p(1_B|\beta_y=m)=\frac{\sum_k p(\alpha_k,1_B)p(\beta_y=m|\alpha_k,1_B)}{p(\beta_y=m)}=\frac{0.25(p_1+p_2)}{0.5(p_1+p_2)}=0.5.\tag{34}$$

Then we need to calculate the conditional error rate $p(1_A|0_B,\beta_y=m)$ and $p(0_A|1_B,\beta_y=m)$. We will divide the situation into two types, namely $m>2\sqrt{T}\Delta a$ and $m<2\sqrt{T}\Delta a$.

When $m>2\sqrt{T}\Delta a$, we can obtain that,

$$p(1_A|0_B,\beta_y=m)=\frac{p(\alpha_0,0_B)p(\beta_y=m|\alpha_0,0_B)}{p(\beta_y=m)p(0_B|\beta_y=m)}=\frac{0.25p_1}{0.25(p_1+p_2)}=\frac{1}{1+e^{\frac{4\sqrt{T}a(m-2\sqrt{T}\Delta a)}{1+T\varepsilon}}}=\frac{1}{1+e^{|\frac{4\sqrt{T}a(m-2\sqrt{T}\Delta a)}{1+T\varepsilon}|}},\tag{35}$$

$$p(0_A|1_B,\beta_y=m)=\frac{p(\alpha_1,1_B)p(\beta_y=m|\alpha_1,1_B)}{p(\beta_y=m)p(1_B|\beta_y=m)}=\frac{0.25p_1}{0.25(p_1+p_2)}=\frac{1}{1+e^{\frac{4\sqrt{T}a(m-2\sqrt{T}\Delta a)}{1+T\varepsilon}}}=\frac{1}{1+e^{|\frac{4\sqrt{T}a(m-2\sqrt{T}\Delta a)}{1+T\varepsilon}|}}.\tag{36}$$

When $m<2\sqrt{T}\Delta a$, we can obtain that,

$$p(1_A|0_B,\beta_y=m)=\frac{p(\alpha_1,0_B)p(\beta_y=m|\alpha_1,0_B)}{p(\beta_y=m)p(0_B|\beta_y=m)}=\frac{0.25p_2}{0.25(p_1+p_2)}=\frac{1}{1+e^{-\frac{4\sqrt{T}a(m-2\sqrt{T}\Delta a)}{1+T\varepsilon}}}=\frac{1}{1+e^{|\frac{4\sqrt{T}a(m-2\sqrt{T}\Delta a)}{1+T\varepsilon}|}},\tag{37}$$

$$p(0_A|1_B,\beta_y=m)=\frac{p(\alpha_0,1_B)p(\beta_y=m|\alpha_0,1_B)}{p(\beta_y=m)p(1_B|\beta_y=m)}=\frac{0.25p_2}{0.25(p_1+p_2)}=\frac{1}{1+e^{-\frac{4\sqrt{T}a(m-2\sqrt{T}\Delta a)}{1+T\varepsilon}}}=\frac{1}{1+e^{|\frac{4\sqrt{T}a(m-2\sqrt{T}\Delta a)}{1+T\varepsilon}|}}.\tag{38}$$

We define that,

$$p_{error}^{BPSK}=1/(1+\exp|\frac{4\sqrt{T}a(m-2\sqrt{T}\Delta a)}{1+T\varepsilon}|).\tag{39}$$

Further, we can obtain that,

$$p(1_A|0_B,\beta_y=m)=p(0_A|1_B,\beta_y=m)=p_{error}^{BPSK}.\tag{40}$$

$$p(0_A|\beta_y=m)=\sum_k p(k_B|\beta_y=m)p(0_A|k_B,\beta_y=m)=0.5,\tag{41}$$

$$p(1_A|\beta_y=m)=\sum_k p(k_B|\beta_y=m)p(1_A|k_B,\beta_y=m)=0.5.\tag{42}$$

Above all, the mutual information between Alice and Bob for BPSK-BE-QKD in this situation is given by,

$$I(A;B|\beta_y=m)=H(A|\beta_y=m)-H(A|B,\beta_y=m)=1-H(p_{error}^{BPSK}).\tag{43}$$

When remaining the modulation constellation unchanged, just changing the detecting method as heterodyne detecting or considering the detection efficiency and the electrical noise, we can just obey the rule discussed before to replace T and ε in the equation (31-43).

For BPSK-BE-QKD, when using the modulation constellation ($\alpha_0=\alpha\exp(i(3\pi/4-\theta))$, $\alpha_1=\alpha\exp(i(-\pi/4-\theta))$) shown in Fig. 8(b) in the main text, the homodyne detection, and ignoring the detection efficiency and the electrical noise, we

can calculate the mutual information between Alice and Bob through following formulas. We can first give out the conditional probabilities of Bob's measurement results,

$$
\begin{aligned}
&p(\beta_y=m|\alpha_0,0_B)=N_{pdf}(m,2\sqrt{T}\Re(\alpha_0),1+T\varepsilon)=p_1,\\
&p(\beta_y=m|\alpha_1,0_B)=N_{pdf}(m,2\sqrt{T}\Re(\alpha_1),1+T\varepsilon)=p_2,\\
&p(\beta_y=m|\alpha_0,1_B)=N_{pdf}(m,2\sqrt{T}\Im(\alpha_0),1+T\varepsilon)=p_3,\\
&p(\beta_y=m|\alpha_1,1_B)=N_{pdf}(m,2\sqrt{T}\Im(\alpha_1),1+T\varepsilon)=p_4.
\end{aligned}
\tag{44}
$$

We can then cluculate that,

$$p(\beta_y=m)=\sum_{k,n}p(\alpha_k,n_B)p(\beta_y=m|\alpha_k,n_B)=0.25(p_1+p_2+p_3+p_4). \tag{45}$$

$$p(0_B|\beta_y=m)=\frac{\sum_k p(\alpha_k,0_B)p(\beta_y=m|\alpha_k,0_B)}{p(\beta_y=m)}=\frac{p_1+p_2}{p_1+p_2+p_3+p_4}, \tag{46}$$

$$p(1_B|\beta_y=m)=\frac{\sum_k p(\alpha_k,1_B)p(\beta_y=m|\alpha_k,1_B)}{p(\beta_y=m)}=\frac{p_3+p_4}{p_1+p_2+p_3+p_4}. \tag{47}$$

Then we need to calculate the conditional error rate $p(1_A|0_B,\beta_y=m)$ and $p(0_A|1_B,\beta_y=m)$. We will divide the situation into two types, namely $0<\theta<\pi/2$ and $-\pi/2<\theta<0$.

When $0<\theta<\pi/2$, we will further divide the situation into three types, namely $m>\sqrt{2T}a\sin\theta$, $-\sqrt{2T}a\sin\theta<m<\sqrt{2T}a\sin\theta$, and $m<-\sqrt{2T}a\sin\theta$.

When $0<\theta<\pi/2$ and $m>\sqrt{2T}a\sin\theta$, we can obtan that,

$$p(1_A|0_B,\beta_y=m)=\frac{p(\alpha_0,0_B)p(\beta_y=m|\alpha_0,0_B)}{p(\beta_y=m)p(0_B|\beta_y=m)}=\frac{p_1}{p_1+p_2}, \tag{48}$$

$$p(0_A|1_B,\beta_y=m)=\frac{p(\alpha_1,1_B)p(\beta_y=m|\alpha_1,1_B)}{p(\beta_y=m)p(1_B|\beta_y=m)}=\frac{p_4}{p_3+p_4}. \tag{49}$$

When $0<\theta<\pi/2$ and $-\sqrt{2T}a\sin\theta<m<\sqrt{2T}a\sin\theta$, we can obtain that,

$$p(1_A|0_B,\beta_y=m)=0, \tag{50}$$

$$p(0_A|1_B,\beta_y=m)=1. \tag{51}$$

When $0<\theta<\pi/2$ and $m<-\sqrt{2T}a\sin\theta$, we can obtain that,

$$p(1_A|0_B,\beta_y=m)=\frac{p(\alpha_1,0_B)p(\beta_y=m|\alpha_1,0_B)}{p(\beta_y=m)p(0_B|\beta_y=m)}=\frac{p_2}{p_1+p_2}, \tag{52}$$

$$p(0_A|1_B,\beta_y=m)=\frac{p(\alpha_0,1_B)p(\beta_y=m|\alpha_0,1_B)}{p(\beta_y=m)p(1_B|\beta_y=m)}=\frac{p_3}{p_3+p_4}. \tag{53}$$

When $-\pi/2<\theta<0$, we will further divide the situation into three types, namely $m>-\sqrt{2T}a\sin\theta$, $\sqrt{2T}a\sin\theta<m<-\sqrt{2T}a\sin\theta$, and $m<\sqrt{2T}a\sin\theta$.

When $-\pi/2<\theta<0$ and $m>-\sqrt{2T}a\sin\theta$, we can obtan that,

$$p(1_A|0_B,\beta_y=m)=\frac{p(\alpha_0,0_B)p(\beta_y=m|\alpha_0,0_B)}{p(\beta_y=m)p(0_B|\beta_y=m)}=\frac{p_1}{p_1+p_2}, \tag{54}$$

$$p(0_A|1_B,\beta_y=m)=\frac{p(\alpha_1,1_B)p(\beta_y=m|\alpha_1,1_B)}{p(\beta_y=m)p(1_B|\beta_y=m)}=\frac{p_4}{p_3+p_4}. \tag{55}$$

When $-\pi/2<\theta<0$ and $\sqrt{2T}a\sin\theta<m<-\sqrt{2T}a\sin\theta$, we can obtain that,

$$p(1_A|0_B,\beta_y=m)=1, \tag{56}$$

$$p(0_A|1_B,\beta_y=m)=0. \tag{57}$$

When $-\pi/2 < \theta < 0$ and $m < \sqrt{2T}a\sin\theta$, we can obtain that,

$$p(1_A|0_B,\beta_y=m)=\frac{p(\alpha_1,0_B)p(\beta_y=m|\alpha_1,0_B)}{p(\beta_y=m)p(0_B|\beta_y=m)}=\frac{p_2}{p_1+p_2}, \tag{58}$$

$$p(0_A|1_B,\beta_y=m)=\frac{p(\alpha_0,1_B)p(\beta_y=m|\alpha_0,1_B)}{p(\beta_y=m)p(1_B|\beta_y=m)}=\frac{p_3}{p_3+p_4}. \tag{59}$$

Furthermore, we can calculate that,

$$p(0_A|\beta_y=m)=\sum_k p(k_B|\beta_y=m)p(0_A|k_B,\beta_y=m), \tag{60}$$

$$p(1_A|\beta_y=m)=\sum_k p(k_B|\beta_y=m)p(1_A|k_B,\beta_y=m). \tag{61}$$

According to formulas (44-61), the mutual information between Alice and Bob for BPSK-BE-QKD in this situation can be calculated by,

$$I(A;B|\beta_y=m)=H(A|\beta_y=m)-H(A|B,\beta_y=m). \tag{62}$$

When remaining the modulation constellation unchanged, just changing the detecting method as heterodyne detecting or considering the detection efficiency and the electrical noise, we can just obey the rule discussed before to replace T and ε in the equations (44-62).

Supplementary Note II: Calculation for the density matrix of conditional quantum state at Eve

In this part, we calculate the density matrix of the quantum state $\rho_E^{x=\lambda_i}$ and $\rho_E^{p=\lambda_i}$ when using the heterodyne detection and ignoring the detection efficiency and the electrical noise, when using the homodyne detection and considering the detection efficiency and the electrical noise, and when using the heterodyne detection and considering the detection efficiency and the electrical noise. The situation of using the homodyne detection and ignoring the detection efficiency and the electrical noise has been discussed in the main text. Supplementary Fig. 1(a) shows the QKD protocol under the entangling cloner attack when using the heterodyne detection and ignoring the detection efficiency and the electrical noise. We can give out the density matrix $\rho_{B_1E_1E_2}$ according to equation (12-15) in the main text. We can also give out the operator of the beam splitter with transmissivity 0.5,

$$R_{G_0B_1}=\exp(\pi/4(\hat{a}_{G_0}\otimes\hat{a}^{\dagger}_{B_1}-\hat{a}^{\dagger}_{G_0}\otimes\hat{a}_{B_1})). \tag{63}$$

Naturally, we can then obtain the density matrix of the state at $B_2E_1E_2$,

$$\rho_{B_2E_1E_2}=Tr_{G_1}((R_{G_0B_1}\otimes I_{E_1E_2})(|0\rangle\langle 0|_{G_0}\otimes\rho_{B_1E_1E_2})(R^{\dagger}_{G_0B_1}\otimes I_{E_1E_2})). \tag{64}$$

Then we can calculate the $\rho_E^{x=\lambda_i}$ and $\rho_E^{p=\lambda_i}$ when using the heterodyne detection and ignoring the detection efficiency and the electrical noise by equations (16-22) in the main text.

Supplementary Fig. 1(b) shows the QKD protocol under the entangling cloner attack when using the homodyne detection and considering the detection efficiency and the electrical noise. According to equations (12-15) in the main text, we can obtain $\rho_{B_1E_1E_2}$. We can also express the operator of the beam splitter with transmissivity η and the state at F_2F_0,

$$R_{F_0B_1}=\exp(\cos^{-1}\sqrt{\eta}(\hat{a}_{F_0}\otimes\hat{a}^{\dagger}_{B_1}-\hat{a}^{\dagger}_{F_0}\otimes\hat{a}_{B_1})), \tag{65}$$

$$|F_2F_0\rangle=\frac{1}{\cosh r_F}\sum_{n=0}^{\infty}(\tanh r_F)^n|n,n\rangle, \tag{66}$$

where $r_F=(\cosh^{-1}V_F)/2$, $V_F=1+v_{el}/(1-\eta)$. Then we can obtain the density matrix of the state at $B_2E_1E_2$,

$$\rho_{B_2E_1E_2}=Tr_{F_2F_1}((I_{F_2}\otimes R_{F_0B_1}\otimes I_{E_1E_2})(|F_2F_0\rangle\langle F_2F_0|\otimes\rho_{B_1E_1E_2})(I_{F_2}\otimes R^{\dagger}_{F_0B_1}\otimes I_{E_1E_2})). \tag{67}$$

We can then calculate the $\rho_E^{x=\lambda_i}$ and $\rho_E^{p=\lambda_i}$ when using the homodyne detection and considering the detection efficiency and the electrical noise by equations (16-22) in the main text.

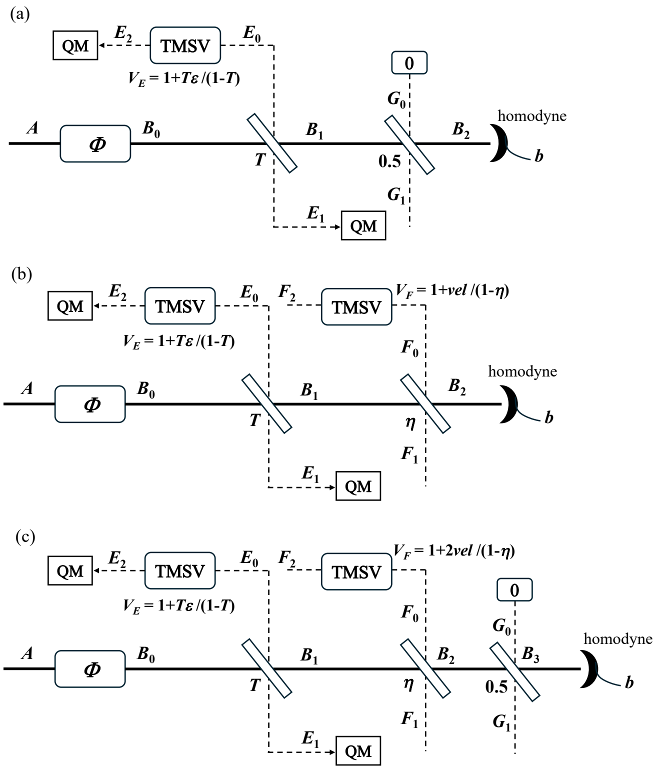

Supplementary Fig. 1. The operation principle of entangling cloner attacks (a) when Bob uses the heterodyne detection while ignoring the detection efficiency and the electrical noise; (b) when Bob uses the homodyne detection while considering the detection efficiency and the electrical noise; (c) when Bob uses the heterodyne detection while considering the detection efficiency and the electrical noise.

Supplementary Fig. 1(c) shows the QKD protocol under the entangling cloner attack when using the heterodyne detection and considering the detection efficiency and the electrical noise. We can obtain $\rho_{B_2E_1E_2}$ according to equations (12-15) in the main text and (65-67). It is worth noting that in this situation, V_F needs to be changed from $1+v_{el}/(1-\eta)$ to $1+2v_{el}/(1-\eta)$. Then we can calculate the $\rho_{B_3E_1E_2}$ using following formulas,

$$R_{G_0B_2} = \exp(\pi/4(\hat{a}_{G_0}\otimes\hat{a}^{\dagger}_{B_2} - \hat{a}^{\dagger}_{G_0}\otimes\hat{a}_{B_2})). \tag{68}$$

$$\rho_{B_3E_1E_2} = Tr_{G_1}((R_{G_0B_2}\otimes I_{E_1E_2})(|0\rangle\langle 0|_{G_0}\otimes\rho_{B_2E_1E_2})(R^{\dagger}_{G_0B_2}\otimes I_{E_1E_2})). \tag{69}$$

We can then calculate the $\rho_E^{x=\lambda_i}$ and $\rho_E^{p=\lambda_i}$ when using the heterodyne detection and considering the detection efficiency and the electrical noise by equations (16-22) in the main text.

Supplementary Note III: Methods for accelerating the calculation

In this part, we derive the expression of $\rho_E^{x=\lambda_i}$ and $\rho_E^{p=\lambda_i}$ to accelerate the speed for calculating $\rho_E^{x=\lambda_i}$ and $\rho_E^{p=\lambda_i}$. We derive the expression of $\rho_E^{x=\lambda_i}$ and $\rho_E^{p=\lambda_i}$ when using the homodyne detection and ignoring the detection efficiency and the electrical noise, when using the heterodyne detection and ignoring the detection efficiency and the electrical noise, when the using homodyne detection and considering the detection efficiency and the electrical noise, and when using the heterodyne detection and considering the detection efficiency and the electrical noise.

1. We first consider the situation that we use the homodyne detection and ignore the detection efficiency and the electrical

noise. We can give out density matrix of the state at $B_1E_1E_2$,

$$\begin{aligned}\rho_{B_1E_1E_2} =&(R_{B_0E_0}\otimes I_{E_2})(\rho_{B_0}\otimes|E_0E_2\rangle\langle E_0E_2|)(R^{\dagger}_{B_0E_0}\otimes I_{E_2}) = (R_{B_0E_0}\otimes I_{E_2})(\sum_{i=0}^{N-1} p_i|\alpha_i\rangle\langle\alpha_i|\otimes|E_0E_2\rangle\langle E_0E_2|)(R^{\dagger}_{B_0E_0}\otimes I_{E_2})\\ =&\sum_{i=0}^{N-1}(p_i(R_{B_0E_0}\otimes I_{E_2})(|\alpha_i\rangle\langle\alpha_i|\otimes|E_0E_2\rangle\langle E_0E_2|)(R^{\dagger}_{B_0E_0}\otimes I_{E_2}))\\ =&\sum_{i=0}^{N-1}(p_i((R_{B_0E_0}\otimes I_{E_2})(|\alpha_i\rangle\otimes|E_0E_2\rangle))((R_{B_0E_0}\otimes I_{E_2})(|\alpha_i\rangle\otimes|E_0E_2\rangle))^{\dagger}).\end{aligned} \tag{70}$$

Then we can define that,

$$|\varphi_i\rangle_{B_1E_1E_2} = |\varphi_i\rangle = (R_{B_0E_0}\otimes I_{E_2})(|\alpha_i\rangle\otimes|E_0E_2\rangle). \tag{71}$$

And we can obtain that,

$$\rho_{B_1E_1E_2} = \sum_{i=0}^{N-1} p_i|\varphi_i\rangle\langle\varphi_i|. \tag{72}$$

Then we can obtain that,

$$\begin{aligned}\rho^{x=\lambda_j}_{B_1E_1E_2} =&\frac{(|x_j\rangle\langle x_j|\otimes I_{E_1E_2})\rho_{B_1E_1E_2}(|x_j\rangle\langle x_j|\otimes I_{E_1E_2})}{Tr((|x_j\rangle\langle x_j|\otimes I_{E_1E_2})(|x_j\rangle\langle x_j|\otimes I_{E_1E_2})\rho_{B_1E_1E_2})} = \frac{\sum_{i=0}^{N-1} p_i(|x_j\rangle\langle x_j|\otimes I_{E_1E_2})|\varphi_i\rangle\langle\varphi_i|(|x_j\rangle\langle x_j|\otimes I_{E_1E_2})}{\sum_{i=0}^{N-1} p_i Tr((|x_j\rangle\langle x_j|\otimes I_{E_1E_2})|\varphi_i\rangle\langle\varphi_i|)}\\ =&\frac{\sum_{i=0}^{N-1} p_i(|x_j\rangle\otimes I_{E_1E_2})(\langle x_j|\otimes I_{E_1E_2})|\varphi_i\rangle\langle\varphi_i|(|x_j\rangle\otimes I_{E_1E_2})(\langle x_j|\otimes I_{E_1E_2})}{\sum_{i=0}^{N-1} p_i Tr(\langle\varphi_i|(|x_j\rangle\langle x_j|\otimes I_{E_1E_2})|\varphi_i\rangle)}\\ =&\frac{\sum_{i=0}^{N-1} p_i(|x_j\rangle\otimes I_{E_1E_2})(\langle x_j|\otimes I_{E_1E_2})|\varphi_i\rangle\langle\varphi_i|(|x_j\rangle\otimes I_{E_1E_2})(\langle x_j|\otimes I_{E_1E_2})}{\sum_{i=0}^{N-1} p_i Tr(\langle\varphi_i|(|x_j\rangle\otimes I_{E_1E_2})(\langle x_j|\otimes I_{E_1E_2})|\varphi_i\rangle)}.\end{aligned} \tag{73}$$

We define that,

$$|\phi_i\rangle_{E_1E_2} = |\phi_i\rangle = (\langle x_j|\otimes I_{E_1E_2})|\varphi_i\rangle, \tag{74}$$

then we can get that,

$$\rho^{x=\lambda_j}_{B_1E_1E_2} = \frac{\sum_{i=0}^{N-1} p_i(|x_j\rangle\otimes I_{E_1E_2})|\phi_i\rangle\langle\phi_i|(\langle x_j|\otimes I_{E_1E_2})}{\sum_{i=0}^{N-1} p_i\langle\phi_i|\phi_i\rangle} = \frac{\sum_{i=0}^{N-1} p_i(|x_j\rangle\langle x_j|\otimes|\phi_i\rangle\langle\phi_i|)}{\sum_{i=0}^{N-1} p_i\langle\phi_i|\phi_i\rangle}, \tag{75}$$

$$\begin{aligned}\rho^{x=\lambda_j}_{E_1E_2} =&Tr_{B_1}(\rho^{x=\lambda_j}_{B_1E_1E_2}) = \frac{\sum_{i=0}^{N-1} p_i Tr_{B_1}(|x_j\rangle\langle x_j|\otimes|\phi_i\rangle\langle\phi_i|)}{\sum_{i=0}^{N-1} p_i\langle\phi_i|\phi_i\rangle}\\ =&\frac{\sum_{i=0}^{N-1} p_i Tr(|x_j\rangle\langle x_j|)|\phi_i\rangle\langle\phi_i|}{\sum_{i=0}^{N-1} p_i\langle\phi_i|\phi_i\rangle} = \frac{\sum_{i=0}^{N-1} p_i Tr(\langle x_j|x_j\rangle)|\phi_i\rangle\langle\phi_i|}{\sum_{i=0}^{N-1} p_i\langle\phi_i|\phi_i\rangle} = \frac{\sum_{i=0}^{N-1} p_i|\phi_i\rangle\langle\phi_i|}{\sum_{i=0}^{N-1} p_i\langle\phi_i|\phi_i\rangle}.\end{aligned} \tag{76}$$

According to the equation (76), $\rho^{x=\lambda_j}_{E_1E_2}$ can be completely calculated by $|\phi_i\rangle$. Meanwhile, the state space of $|\phi_i\rangle$ is E_1E_2. So, if we can calculate $|\phi_i\rangle$, then we can easily get $\rho^{x=\lambda_j}_{E_1E_2}$ by the equation (76) and avoid the calculation involving the sate space of

$B_1E_1E_2$. The required computing resources and time have been greatly reduced. So, we will start to calculate the $|\phi_i\rangle$ next. We know that $|\alpha_i\rangle = e^{-\frac{|\alpha_i|^2}{2}} \sum_{n=0}^{\infty} \frac{\alpha_i^n}{\sqrt{n!}} |n\rangle$, then we can obtain that,

$$|\alpha_i\rangle \otimes |E_0E_2\rangle = \frac{e^{-\frac{|\alpha_i|^2}{2}}}{\cosh r_E} \sum_{n,m} (\tanh r_E)^m \frac{\alpha_i^n}{\sqrt{n!}} |n,m,m\rangle . \tag{77}$$

We define $\hat{a}$ as the annihilation operation at B_0 and $\hat{b}$ as the annihilation operation at E_0, then we can obtain that,

$$|\alpha_i\rangle \otimes |E_0E_2\rangle = \frac{e^{-\frac{|\alpha_i|^2}{2}}}{\cosh r_E} \sum_{n,m} (\tanh r_E)^m \frac{\alpha_i^n}{\sqrt{n!}} \frac{(\hat{a}^\dagger)^n}{\sqrt{n!}} \frac{(\hat{b}^\dagger)^m}{\sqrt{m!}} |0,0\rangle \otimes |m\rangle = \sum_m \frac{e^{-\frac{|\alpha_i|^2}{2}} (\tanh r_E)^m}{\cosh r_E \sqrt{m!}} (\sum_n \frac{\alpha_i^n}{n!} (\hat{a}^\dagger)^n (\hat{b}^\dagger)^m |0,0\rangle) \otimes |m\rangle . \tag{78}$$

Accordint to the input-output relationship of the beam splitter, we know that,

$$\begin{aligned} \hat{a}_{out} &= \sqrt{T}\hat{a}_{in} + \sqrt{1-T}\hat{b}_{in} \\ \hat{b}_{out} &= -\sqrt{1-T}\hat{a}_{in} + \sqrt{T}\hat{b}_{in}. \end{aligned} \tag{79}$$

We can then calculate that,

$$\begin{aligned} |\varphi_i\rangle &= (R_{B_0E_0} \otimes I_{E_2})(|\alpha_i\rangle \otimes |E_0E_2\rangle) \\ &= \sum_m \frac{e^{-\frac{|\alpha_i|^2}{2}} (\tanh r_E)^m}{\cosh r_E \sqrt{m!}} (\sum_n \frac{\alpha_i^n}{n!} (\sqrt{T}\hat{a}^\dagger + \sqrt{1-T}\hat{b}^\dagger)^n (-\sqrt{1-T}\hat{a}^\dagger + \sqrt{T}\hat{b}^\dagger)^m |0,0\rangle) \otimes |m\rangle . \end{aligned} \tag{80}$$

We define that,

$$|\chi_i\rangle = \sum_n \frac{\alpha_i^n}{n!} (\sqrt{T}\hat{a}^\dagger + \sqrt{1-T}\hat{b}^\dagger)^n (-\sqrt{1-T}\hat{a}^\dagger + \sqrt{T}\hat{b}^\dagger)^m |0,0\rangle , \tag{81}$$

and then we can obtain that,

$$|\varphi_i\rangle = \sum_m \frac{e^{-\frac{|\alpha_i|^2}{2}} (\tanh r_E)^m}{\cosh r_E \sqrt{m!}} |\chi_i\rangle \otimes |m\rangle . \tag{82}$$

We can further obtain that,

$$\begin{aligned} |\chi_i\rangle &= \sum_n \frac{\alpha_i^n}{n!} (\sqrt{T}\hat{a}^\dagger + \sqrt{1-T}\hat{b}^\dagger)^n (-\sqrt{1-T}\hat{a}^\dagger + \sqrt{T}\hat{b}^\dagger)^m |0,0\rangle \\ &= \sum_n \frac{\alpha_i^n}{n!} (\sum_{k=0}^{n} C_n^k (\sqrt{T}\hat{a}^\dagger)^k (\sqrt{1-T}\hat{b}^\dagger)^{n-k}) (\sum_{k=0}^{m} C_m^k (-\sqrt{1-T}\hat{a}^\dagger)^k (\sqrt{T}\hat{b}^\dagger)^{m-k}) |0,0\rangle \\ &= \sum_{n=0}^{\infty} \sum_{k_1=0}^{n} \sum_{k_2=0}^{m} \frac{\alpha_i^n}{n!} C_n^{k_1} (\sqrt{T}\hat{a}^\dagger)^{k_1} (\sqrt{1-T}\hat{b}^\dagger)^{n-k_1} C_m^{k_2} (-\sqrt{1-T}\hat{a}^\dagger)^{k_2} (\sqrt{T}\hat{b}^\dagger)^{m-k_2} |0,0\rangle \\ &= \sum_{n=0}^{\infty} \sum_{k_1=0}^{n} \sum_{k_2=0}^{m} (-1)^{k_2} \frac{\alpha_i^n}{n!} C_n^{k_1} C_m^{k_2} (\sqrt{T})^{m+k_1-k_2} (\sqrt{1-T})^{n+k_2-k_1} \sqrt{(k_1+k_2)!} \sqrt{(n+m-k_1-k_2)!} |k_1+k_2, n+m-k_1-k_2\rangle . \end{aligned} \tag{83}$$

We make $k_1 + k_2 = x_1$, $n + m - k_1 - k_2 = x_2$, and that,

$$|\chi_i\rangle = \sum_{x_1,x_2} \psi_i(x_1,x_2,m) |x_1,x_2\rangle . \tag{84}$$

Considering $n \in [0,\infty)$, $k_1 \in [0,n]$ and $k_2 \in [0,m]$, we can obtain that,

$$\begin{aligned} 0 \le n = x_1 + x_2 - m &\Rightarrow x_1 + x_2 \ge m \\ 0 \le k_1 \le n = x_1 + x_2 - m &\Rightarrow 0 \le k_1 \le x_1 + x_2 - m \\ 0 \le k_2 = x_1 - k_1 \le m &\Rightarrow x_1 - m \le k_1 \le x_1 . \end{aligned} \tag{85}$$

Then we can get,

$$\psi_i(x_1,x_2,m)$$
$$=\begin{cases}\sum_{k_1=\max(0,x_1-m)}^{\min(x_1,x_1+x_2-m)}((-1)^{x_1-k_1}\frac{\alpha_i^{x_1+x_2-m}}{(x_1+x_2-m)!}C_{x_1+x_2-m}^{k_1}C_m^{x_1-k_1} & ,x_1+x_2\geq m\\ \times(\sqrt{T})^{m+2k_1-x_1}(\sqrt{1-T})^{2x_1+x_2-m-2k_1}\sqrt{x_1!}\sqrt{x_2!}) & \\ 0 & ,x_1+x_2<m\end{cases}. \tag{86}$$

We can further solve and simplify the above equation to obtain,

$$\psi_i(x_1,x_2,m)$$
$$=\begin{cases}\frac{(-1)^m\alpha_i^{x_1+x_2-m}(\sqrt{T})^{x_1-m}(\sqrt{1-T})^{x_2+m}\sqrt{x_1!}\,{}_2F_1(-m,-x_2;1-m+x_1;\frac{T}{T-1})}{(x_1-m)!\sqrt{x_2!}} & ,(x_1+x_2\geq m)\&(x_1>m)\\ \frac{(-1)^{x_1}\alpha_i^{x_1+x_2-m}(\sqrt{T})^{m-x_1}(\sqrt{1-T})^{2x_1+x_2-m}m!\sqrt{x_2!}}{(m-x_1)!(x_1+x_2-m)!\sqrt{x_1!}} & ,(x_1+x_2\geq m)\&(x_1\leq m)\\ \times{}_2F_1(-x_1,m-x_1-x_2;1+m-x_1;\frac{T}{T-1}) & \\ 0 & ,x_1+x_2<m\end{cases}, \tag{87}$$

where ${}_2F_1(a,b;c;x)$ is the hypergeometric function. We can further define that,

$$\zeta_i(x_1,x_2,m)=\frac{e^{-\frac{|\alpha_i|^2}{2}}(\tanh r_E)^m}{\cosh r_E\sqrt{m!}}\psi_i(x_1,x_2,m). \tag{88}$$

Then we can obtain,

$$\begin{aligned}|\varphi_i\rangle =&\sum_m\frac{e^{-\frac{|\alpha_i|^2}{2}}(\tanh r_E)^m}{\cosh r_E\sqrt{m!}}|\chi_i\rangle\otimes|m\rangle=\sum_m\frac{e^{-\frac{|\alpha_i|^2}{2}}(\tanh r_E)^m}{\cosh r_E\sqrt{m!}}(\sum_{x_1,x_2}\psi_i(x_1,x_2,m)|x_1,x_2\rangle)\otimes|m\rangle\\ =&\sum_{x_1,x_2,m}\frac{e^{-\frac{|\alpha_i|^2}{2}}(\tanh r_E)^m}{\cosh r_E\sqrt{m!}}\psi_i(x_1,x_2,m)|x_1,x_2,m\rangle=\sum_{x_1,x_2,m}\zeta_i(x_1,x_2,m)|x_1,x_2,m\rangle.\end{aligned} \tag{89}$$

We define that,

$$|x_j\rangle=\sum_y\gamma_{x_j}(y)|y\rangle, \tag{90}$$

then can get that,

$$\begin{aligned}|\phi_i\rangle =&(\langle x_j|\otimes I_{E_1E_2})|\varphi_i\rangle=((\sum_y\gamma_{x_j}(y)|y\rangle)^\dagger\otimes I_{E_1E_2})(\sum_{x_1,x_2,m}\zeta_i(x_1,x_2,m)|x_1,x_2,m\rangle)\\ =&\sum_{y,x_1,x_2,m}\bar{\gamma}_{x_j}\zeta_i(x_1,x_2,m)(\langle y|\otimes I_{E_1E_2})(|x_1\rangle\otimes|x_2,m\rangle)=\sum_{y,x_1,x_2,m}\bar{\gamma}_{x_j}\zeta_i(x_1,x_2,m)(\langle y|x_1\rangle\otimes|x_2,m\rangle)\\ =&\sum_{x_1,x_2,m}\bar{\gamma}_{x_j}\zeta_i(x_1,x_2,m)|x_2,m\rangle=\sum_{x_2,m}(\sum_{x_1}\bar{\gamma}_{x_j}\zeta_i(x_1,x_2,m))|x_2,m\rangle=\sum_{n_1,n_2}(\sum_{x_1}\bar{\gamma}_{x_j}\zeta_i(x_1,n_1,n_2))|n_1,n_2\rangle.\end{aligned} \tag{91}$$

We then define that,

$$\kappa_{x_j}(n_1,n_2)=\sum_{x_1}\bar{\gamma}_{x_j}\zeta_i(x_1,n_1,n_2). \tag{92}$$

Finally, we can obtain that,

$$\left|\phi_i\right\rangle = \sum_{n_1,n_2} \kappa_{x_j}(n_1,n_2)\left|n_1,n_2\right\rangle. \tag{93}$$

According to equations (76), (87-88), (90) and (92-93), we can easily calculate $\rho_{E_1E_2}^{x=\lambda_j}$. Similarly, we define $|p_j\rangle = \sum_y \gamma_{p_j}(y)\,|y\rangle$ and can use the following equations to calculate $\rho_{E_1E_2}^{p=\lambda_j}$,

$$\rho_{E_1E_2}^{p=\lambda_j} = \frac{\sum_{i=0}^{N-1} p_i|\phi_i\rangle\langle\phi_i|}{\sum_{i=0}^{N-1} p_i\langle\phi_i|\phi_i\rangle}, \tag{94}$$

$$\left|\phi_i\right\rangle = \sum_{n_1,n_2} \kappa_{p_j}(n_1,n_2)\left|n_1,n_2\right\rangle, \tag{95}$$

$$\kappa_{p_j}(n_1,n_2) = \sum_{x_1} \bar{\gamma}_{p_j}\zeta_i(x_1,n_1,n_2). \tag{96}$$

2. We then consider the situation that we use the heterodyne detection and ignore the detection efficiency and the electrical noise. According to equations (70-72), we can obtain the density matrix of the state at $B_1E_1E_2$. Then, the state after passing through a beam splitter with transmissivity 0.5 can be given by,

$$\begin{aligned}\rho_{G_1B_2E_1E_2} &= (R_{G_0B_1}\otimes I_{E_1E_2})(|0\rangle\langle 0|_{G_0}\otimes\rho_{B_1E_1E_2})(R_{G_0B_1}^{\dagger}\otimes I_{E_1E_2}) = \sum_{i=0}^{N-1}(p_i(R_{G_0B_1}\otimes I_{E_1E_2})(|0\rangle\langle 0|_{G_0}\otimes|\varphi_i\rangle\langle\varphi_i|)(R_{G_0B_1}^{\dagger}\otimes I_{E_1E_2}))\\ &= \sum_{i=0}^{N-1}(p_i((R_{G_0B_1}\otimes I_{E_1E_2})(|0\rangle\otimes|\varphi_i\rangle))((R_{G_0B_1}\otimes I_{E_1E_2})(|0\rangle\otimes|\varphi_i\rangle))^{\dagger}).\end{aligned} \tag{97}$$

We can define that

$$|\Gamma_i\rangle_{G_1B_2E_1E_2} = |\Gamma_i\rangle = (R_{G_0B_1}\otimes I_{E_1E_2})(|0\rangle\otimes|\varphi_i\rangle), \tag{98}$$

and then can obtain that,

$$\rho_{B_2E_1E_2} = Tr_{G_1}(\rho_{G_1B_2E_1E_2}) = Tr_{G_1}(\sum_{i=0}^{N-1} p_i|\Gamma_i\rangle\langle\Gamma_i|) = \sum_{i=0}^{N-1} p_i Tr_{G_1}(|\Gamma_i\rangle\langle\Gamma_i|). \tag{99}$$

Then, we can obtain that,

$$\begin{aligned}\rho_{B_2E_1E_2}^{x=\lambda_j} &= \frac{(|x_j\rangle\langle x_j|\otimes I_{E_1E_2})\rho_{B_2E_1E_2}(|x_j\rangle\langle x_j|\otimes I_{E_1E_2})}{Tr((|x_j\rangle\langle x_j|\otimes I_{E_1E_2})(|x_j\rangle\langle x_j|\otimes I_{E_1E_2})\rho_{B_2E_1E_2})} = \frac{(|x_j\rangle\langle x_j|\otimes I_{E_1E_2})\rho_{B_2E_1E_2}(|x_j\rangle\langle x_j|\otimes I_{E_1E_2})}{Tr((|x_j\rangle\langle x_j|\otimes I_{E_1E_2})\rho_{B_2E_1E_2})}\\ &= \frac{(|x_j\rangle\otimes I_{E_1E_2})(((\langle x_j|\otimes I_{E_1E_2})\rho_{B_2E_1E_2}(|x_j\rangle\otimes I_{E_1E_2}))(\langle x_j|\otimes I_{E_1E_2})}{Tr((\langle x_j|\otimes I_{E_1E_2})\rho_{B_2E_1E_2}(|x_j\rangle\otimes I_{E_1E_2}))}.\end{aligned} \tag{100}$$

We can further define that,

$$\begin{aligned}\rho_{E_1E_2}^{x=\lambda_j} &= Tr_{B_2}(\rho_{B_1E_1E_2}^{x=\lambda_i}) = \frac{\sum_i((\langle i|x_j\rangle\otimes I_{E_1E_2})(((\langle x_j|\otimes I_{E_1E_2})\rho_{B_2E_1E_2}(|x_j\rangle\otimes I_{E_1E_2}))(\langle x_j|i\rangle\otimes I_{E_1E_2}))}{Tr((\langle x_j|\otimes I_{E_1E_2})\rho_{B_2E_1E_2}(|x_j\rangle\otimes I_{E_1E_2}))}\\ &= \frac{\sum_i((\langle x_j|x_j\rangle\otimes I_{E_1E_2})(((\langle x_j|\otimes I_{E_1E_2})\rho_{B_2E_1E_2}(|x_j\rangle\otimes I_{E_1E_2})))}{Tr((\langle x_j|\otimes I_{E_1E_2})\rho_{B_2E_1E_2}\times(|x_j\rangle\otimes I_{E_1E_2}))} = \frac{(\langle x_j|\otimes I_{E_1E_2})\rho_{B_2E_1E_2}(|x_j\rangle\otimes I_{E_1E_2})}{Tr((\langle x_j|\otimes I_{E_1E_2})\rho_{B_2E_1E_2}(|x_j\rangle\otimes I_{E_1E_2}))},\end{aligned} \tag{101}$$

then we define that,

$$\rho_{x_j,\Xi}^{E_1E_2} = (\langle x_j|\otimes I_{E_1E_2})\rho_{B_2E_1E_2}(|x_j\rangle\otimes I_{E_1E_2}), \tag{102}$$

and we can then obtain,

$$\rho_{E_1E_2}^{x=\lambda_j} = \rho_{x_j,\Xi}^{E_1E_2}/Tr(\rho_{x_j,\Xi}^{E_1E_2}). \tag{103}$$

According to the equation (103), $\rho_{E_1E_2}^{x=\lambda_j}$ can be completely calculated by $\rho_{x_j,\Xi}^{E_1E_2}$. Meanwhile, the state space of $\rho_{x_j,\Xi}^{E_1E_2}$ is E_1E_2. So, if we can calculate $\rho_{x_j,\Xi}^{E_1E_2}$, then we can easily get $\rho_{E_1E_2}^{x=\lambda_j}$ by the equation (103) and avoid the calculation involving the sate space of $B_1E_1E_2$, which can accelerate the calculation speed. So, we will start to calculate the $\rho_{x_j,\Xi}^{E_1E_2}$ next. According to equations (87-89), we can get the expression of the $|\varphi_i\rangle$ and can further obtain that,

$$|0\rangle \otimes |\varphi_i\rangle = \sum_{n,y_1,y_2} \zeta_i(n,y_1,y_2)\,|0,n,y_1,y_2\rangle\,. \tag{104}$$

We define $\hat{a}$ as the annihilation operation at G_0 and $\hat{b}$ as the annihilation operation at B_1, then we can obtain that,

$$|0\rangle \otimes |\varphi_i\rangle = \sum_{y_1,y_2} (\sum_{n} \zeta_i(n,y_1,y_2)\frac{(\hat{b}^\dagger)^n}{\sqrt{n!}}\,|0,0\rangle)\otimes|y_1,y_2\rangle\,. \tag{105}$$

According to the input-output relationship of the beam splitter and equation (98), we can calculate that,

$$\begin{aligned}
|\Gamma_i\rangle =&(R_{G_0B_1}\otimes I_{E_1E_2})(|0\rangle\otimes|\varphi_i\rangle) = \sum_{y_1,y_2}((\sum_{n}\zeta_i(n,y_1,y_2)\frac{(-\sqrt{0.5}\hat{a}^\dagger+\sqrt{0.5}\hat{b}^\dagger)^n}{\sqrt{n!}}\,|0,0\rangle)\otimes|y_1,y_2\rangle)\\
&=\sum_{y_1,y_2}((\sum_{n}\zeta_i(n,y_1,y_2)/\sqrt{n!}(\sum_{k=0}^{n}C_n^k(-\sqrt{0.5}\hat{a}^\dagger)^k(\sqrt{0.5}\hat{b}^\dagger)^{n-k}\,|0,0\rangle))\otimes|y_1,y_2\rangle)\\
&=\sum_{y_1,y_2}((\sum_{n,k}\zeta_i(n,y_1,y_2)/\sqrt{n!}\cdot C_n^k(-1)^k(\sqrt{0.5})^n(\hat{a}^\dagger)^k(\hat{a}^\dagger)^{n-k}\,|0,0\rangle)\otimes|y_1,y_2\rangle)\\
&=\sum_{y_1,y_2}((\sum_{n,k}\zeta_i(n,y_1,y_2)/\sqrt{n!}\cdot C_n^k(-1)^k(\sqrt{0.5})^n\sqrt{k!}\sqrt{(n-k)!}\,|k,n-k\rangle)\otimes|y_1,y_2\rangle).
\end{aligned} \tag{106}$$

Then we define $x_1 = k$, $x_2 = n-k$ and can further obtain that,

$$|\Gamma_i\rangle = \sum_{x_1,x_2,y_1,y_2} \zeta_i(x_1+x_2,y_1,y_2)/\sqrt{(x_1+x_2)!}\cdot C_{x_1+x_2}^{x_1}(-1)^{x_1}(\sqrt{0.5})^{x_1+x_2}\sqrt{x_1!}\sqrt{x_2!}\,|x_1,x_2,y_1,y_2\rangle\,. \tag{107}$$

We define that,

$$\tau_i(x_1,x_2,y_1,y_2) = \zeta_i(x_1+x_2,y_1,y_2)/\sqrt{(x_1+x_2)!}C_{x_1+x_2}^{x_1}(-1)^{x_1}(\sqrt{0.5})^{x_1+x_2}\sqrt{x_1!}\sqrt{x_2!}. \tag{108}$$

We can then obtain that,

$$|\Gamma_i\rangle = \sum_{x_1,x_2,y_1,y_2} \tau_i(x_1,x_2,y_1,y_2)\,|x_1,x_2,y_1,y_2\rangle\,, \tag{109}$$

$$\begin{aligned}
Tr_{G_1}(|\Gamma_i\rangle\langle\Gamma_i|) =&\sum_{x_1}(\langle x_1|\otimes I_{B_2E_1E_2})|\Gamma_i\rangle\langle\Gamma_i|(|x_1\rangle\otimes I_{B_2E_1E_2})\\
=&\sum_{x_1,x_2,x_3,y_1,y_2,y_3,y_4}\tau_i(x_1,x_2,y_1,y_2)\bar{\tau}_i(x_1,x_3,y_3,y_4)|x_2,y_1,y_2\rangle\langle x_3,y_3,y_4|\\
=&\sum_{x_2,x_3,y_1,y_2,y_3,y_4}(\sum_{x_1}\tau_i(x_1,x_2,y_1,y_2)\bar{\tau}_i(x_1,x_3,y_3,y_4))|x_2,y_1,y_2\rangle\langle x_3,y_3,y_4|.
\end{aligned} \tag{110}$$

Then we define that,

$$\Phi_i(x_2,y_1,y_2,x_3,y_3,y_4) = \sum_{x_1}\tau_i(x_1,x_2,y_1,y_2)\bar{\tau}_i(x_1,x_3,y_3,y_4), \tag{111}$$

and we can obtain that,

$$\rho_{B_2E_1E_2} = \sum_{i=0}^{N-1} p_i Tr_{G_1}(|\Gamma_i\rangle\langle\Gamma_i|) = \sum_{i=0}^{N-1} p_i\Phi_i(x_2,y_1,y_2,x_3,y_3,y_4)|x_2,y_1,y_2\rangle\langle x_3,y_3,y_4|. \tag{112}$$

We further define that,

$$\Phi(x_2,y_1,y_2,x_3,y_3,y_4) = \sum_{i=0}^{N-1} p_i\Phi_i(x_2,y_1,y_2,x_3,y_3,y_4), \tag{113}$$

and can naturally get that,

$$\rho_{B_2E_1E_2} = \Phi(x_2,y_1,y_2,x_3,y_3,y_4)|x_2,y_1,y_2\rangle\langle x_3,y_3,y_4|, \tag{114}$$

$$\begin{aligned}
\rho^{E_1E_2}_{x=\lambda_j,\Xi} =&(\langle x_j|\otimes I_{E_1E_2})\rho_{B_2E_1E_2}(|x_j\rangle\otimes I_{E_1E_2})\\
=&((\sum_y \gamma_{x_j}(y)\,|y\rangle)^\dagger\otimes I_{E_1E_2})(\sum_{x_2,x_3,y_1,y_2,y_3,y_4}\Phi(x_2,y_1,y_2,x_3,y_3,y_4)|x_2,y_1,y_2\rangle\langle x_3,y_3,y_4|)((\sum_y \gamma_{x_j}(y)\,|y\rangle)\otimes I_{E_1E_2})\\
=&((\sum_y \bar{\gamma}_{x_j}(y)\,\langle y|)\otimes I_{E_1E_2})(\sum_{x_2,x_3,y_1,y_2,y_3,y_4}\Phi(x_2,y_1,y_2,x_3,y_3,y_4)|x_2,y_1,y_2\rangle\langle x_3,y_3,y_4|)((\sum_y \gamma_{x_j}(y)\,|y\rangle)\otimes I_{E_1E_2})\\
=&\sum_{y_1,y_2,y_3,y_4}(\sum_{x_2,x_3}\bar{\gamma}_{x_j}(x_2)\gamma_{x_j}(x_3)\Phi(x_2,y_1,y_2,x_3,y_3,y_4))|y_1,y_2\rangle\langle y_3,y_4|.
\end{aligned} \tag{115}$$

We define that,

$$\Theta(y_1,y_2,y_3,y_4) = \sum_{x_2,x_3}\bar{\gamma}_{x_j}(x_2)\gamma_{x_j}(x_3)\Phi(x_2,y_1,y_2,x_3,y_3,y_4). \tag{116}$$

We can then obtain that,

$$\rho^{E_1E_2}_{x=\lambda_j,\Xi} = \sum_{y_1,y_2,y_3,y_4}\Theta(y_1,y_2,y_3,y_4)|y_1,y_2\rangle\langle y_3,y_4|. \tag{117}$$

According to equations (87-88), (103), (108), (111), (113) and (116-117), we can easily calculate $\rho^{x=\lambda_i}_{E_1E_2}$. Similarly, we can use following equations to calculate $\rho^{p=\lambda_i}_{E_1E_2}$,

$$\rho^{p=\lambda_j}_{E_1E_2} = \rho^{E_1E_2}_{p_j,\Xi}/Tr(\rho^{E_1E_2}_{p_j,\Xi}), \tag{118}$$

$$\rho^{E_1E_2}_{p=\lambda_j,\Xi} = \sum_{y_1,y_2,y_3,y_4}\Theta(y_1,y_2,y_3,y_4)|y_1,y_2\rangle\langle y_3,y_4|, \tag{119}$$

$$\Theta(y_1,y_2,y_3,y_4) = \sum_{x_2,x_3}\bar{\gamma}_{p_j}(x_2)\gamma_{p_j}(x_3)\Phi(x_2,y_1,y_2,x_3,y_3,y_4). \tag{120}$$

3. We then consider the situation that we use the homodyne detection and consider the detection efficiency and the electrical noise. According to equations (70-72), we can obtain the density matrix of the state at $B_1E_1E_2$. Then, the state after passing through a beam splitter with transmissivity η can be given by,

$$\begin{aligned}
\rho_{F_2F_1B_2E_1E_2} =&(I_{F_2}\otimes R_{F_0B_1}\otimes I_{E_1E_2})(|F_2F_0\rangle\langle F_2F_0|\otimes\rho_{B_1E_1E_2})(I_{F_2}\otimes R^\dagger_{F_0B_1}\otimes I_{E_1E_2})\\
=&\sum_{i=0}^{N-1}(p_i(I_{F_2}\otimes R_{F_0B_1}\otimes I_{E_1E_2})(|F_2F_0\rangle\langle F_2F_0|\otimes|\varphi_i\rangle\langle\varphi_i|)(I_{F_2}\otimes R^\dagger_{F_0B_1}\otimes I_{E_1E_2}))\\
=&\sum_{i=0}^{N-1}(p_i((I_{F_2}\otimes R_{F_0B_1}\otimes I_{E_1E_2})(|F_2F_0\rangle\otimes|\varphi_i\rangle))((I_{F_2}\otimes R_{F_0B_1}\otimes I_{E_1E_2})(|F_2F_0\rangle\otimes|\varphi_i\rangle))^\dagger).
\end{aligned} \tag{121}$$

We define that,

$$|\delta_i\rangle_{F_2F_1B_2E_1E_2} = |\delta_i\rangle = (I_{F_2}\otimes R_{F_0B_1}\otimes I_{E_1E_2})(|F_2F_0\rangle\otimes|\varphi_i\rangle), \tag{122}$$

and we can further obtain,

$$\rho_{F_2F_1B_2E_1E_2} = \sum_{i=0}^{N-1}p_i|\delta_i\rangle\langle\delta_i|, \tag{123}$$

$$\rho_{B_2E_1E_2} = Tr_{F_2F_1}(\rho_{F_2F_1B_2E_1E_2}) = \sum_{i=0}^{N-1}p_iTr_{F_2F_1}(|\delta_i\rangle\langle\delta_i|). \tag{124}$$

Similar to the above derivations, we can use equation (103) (where $\rho^{E_1E_2}_{x_j,\Xi} = (\langle x_j|\otimes I_{E_1E_2})\rho_{B_2E_1E_2}(|x_j\rangle\otimes I_{E_1E_2})$) to calculate $\rho^{x=\lambda_i}_{E_1E_2}$ with the higher calculating speed. So, we will start to calculate the $\rho^{E_1E_2}_{x_j,\Xi}$ in this situation next. According to equations (87-89), we can get the expresssion of the $|\varphi_i\rangle$ and can further obtain that,

$$|F_2F_0\rangle\otimes|\varphi_i\rangle = \frac{1}{\cosh r_F}\sum_{m,n,y_1,y_2}(\tanh r_F)^m\zeta_i(n,y_1,y_2)\,|m,m,n,y_1,y_2\rangle\,. \tag{125}$$

We define $\hat{a}$ as the annihilation operator at F_0 and $\hat{b}$ as the annihilation operator at B_1, then we can obtain that,

$$\begin{aligned}|F_2F_0\rangle\otimes|\varphi_i\rangle &= \sum_{m,y_1,y_2}(\frac{(\tanh r_F)^m}{\cosh r_F}|m\rangle\otimes(\sum_n \frac{(\hat{a}^\dagger)^m}{\sqrt{m!}}\frac{(\hat{b}^\dagger)^n}{\sqrt{n!}}\zeta_i(n,y_1,y_2)|0,0\rangle)\otimes|y_1,y_2\rangle) \\ &= \sum_{m,y_1,y_2}(\frac{(\tanh r_F)^m}{\cosh r_F\sqrt{m!}}|m\rangle\otimes(\sum_n \zeta_i(n,y_1,y_2)/\sqrt{n!}(\hat{a}^\dagger)^m(\hat{b}^\dagger)^n|0,0\rangle)\otimes|y_1,y_2\rangle).\end{aligned} \tag{126}$$

According to the input-output relationship of the beam splitter, we can calculate that,

$$\begin{aligned}|\delta_i\rangle =&(I_{F_2}\otimes R_{F_0B_1}\otimes I_{E_1E_2})(|F_2F_0\rangle\otimes|\varphi_i\rangle) \\ =&\sum_{m,y_1,y_2}\frac{(\tanh r_F)^m}{\cosh r_F\sqrt{m!}}|m\rangle\otimes(\sum_n\zeta_i(n,y_1,y_2)/\sqrt{n!}(-\sqrt{1-\eta}\hat{a}^\dagger+\sqrt{\eta}\hat{b}^\dagger)^n(\sqrt{\eta}\hat{a}^\dagger+\sqrt{1-\eta}\hat{b}^\dagger)^m|0,0\rangle)\otimes|y_1,y_2\rangle \\ =&\sum_{m,y_1,y_2}\frac{(\tanh r_F)^m}{\cosh r_F\sqrt{m!}}|m\rangle\otimes(\sum_n\zeta_i(n,y_1,y_2)/\sqrt{n!}(\sum_{k=0}^{n}C_n^k(-\sqrt{1-\eta}\hat{a}^\dagger)^k \\ &\times(\sqrt{\eta}\hat{b}^\dagger)^{n-k})(\sum_{k=0}^{m}C_m^k(\sqrt{\eta}\hat{a}^\dagger)^k(\sqrt{1-\eta}\hat{b}^\dagger)^{m-k})|0,0\rangle)\otimes|y_1,y_2\rangle \\ =&\sum_{m,y_1,y_2}\frac{(\tanh r_F)^m}{\cosh r_F\sqrt{m!}}|m\rangle\otimes(\sum_{n,k_1,k_2}\zeta_i(n,y_1,y_2)/\sqrt{n!}C_n^{k_1}C_m^{k_2}(-1)^{k_1} \\ &\times(\sqrt{1-\eta})^{k_1+m-k_2}(\sqrt{\eta})^{n-k_1+k_2}(\hat{a}^\dagger)^{k_1+k_2}(\hat{b}^\dagger)^{n+m-k_1-k_2}|0,0\rangle)\otimes|y_1,y_2\rangle \\ =&\sum_{m,y_1,y_2}\frac{(\tanh r_F)^m}{\cosh r_F\sqrt{m!}}|m\rangle\otimes(\sum_{n,k_1,k_2}\zeta_i(n,y_1,y_2)/\sqrt{n!}C_n^{k_1}C_m^{k_2}(-1)^{k_1} \\ &\times(\sqrt{1-\eta})^{k_1+m-k_2}(\sqrt{\eta})^{n-k_1+k_2}\sqrt{(k_1+k_2)!}\sqrt{(n+m-k_1-k_2)!}|k_1+k_2,n+m-k_1-k_2\rangle)\otimes|y_1,y_2\rangle.\end{aligned} \tag{127}$$

We make $k_1+k_2=x_1$, $n+m-k_1-k_2=x_2$. Considering $n\in[0,\infty)$, $k_1\in[0,n]$ and $k_2\in[0,m]$, we can obtain that,

$$\begin{aligned}0\le n=x_1+x_2-m&\Rightarrow x_1+x_2\ge m \\ 0\le k_1\le n=x_1+x_2-m&\Rightarrow 0\le k_1\le x_1+x_2-m \\ 0\le k_2=x_1-k_1\le m&\Rightarrow x_1-m\le k_1\le x_1.\end{aligned} \tag{128}$$

Then we can get that,

$$\begin{aligned}|\delta_i\rangle =& \sum_{m,x_1,x_2,y_1,y_2,x_1+x_2\ge m}\frac{(\tanh r_F)^m\sqrt{x_1!}\sqrt{x_2!}}{\cosh r_F\sqrt{m!}\sqrt{(x_1+x_2-m)!}}\zeta_i(x_1+x_2-m,y_1,y_2)|m\rangle \\ &\otimes(\sum_{k_1=\max(0,x_1-m)}^{\min(x_1,x_1+x_2-m)}(-1)^{k_1}C_{x_1+x_2-m}^{k_1}C_m^{x_1-k_1}(\sqrt{1-\eta})^{2k_1+m-x_1}(\sqrt{\eta})^{2x_1+x_2-m-2k_1}|x_1,x_2\rangle)\otimes|y_1,y_2\rangle.\end{aligned} \tag{129}$$

We then define that,

$$\vartheta_i(x_1,x_2,m)=\begin{cases}\sum_{k_1=\max(0,x_1-m)}^{\min(x_1,x_1+x_2-m)}(-1)^{k_1}C_{x_1+x_2-m}^{k_1}C_m^{x_1-k_1}(\sqrt{1-\eta})^{2k_1+m-x_1}(\sqrt{\eta})^{2x_1+x_2-m-2k_1} & ,x_1+x_2\ge m \\ 0 & ,x_1+x_2<m\end{cases}. \tag{130}$$

We can further solve and simplify the above equation to obtain,

$$\begin{aligned}&\vartheta(x_1,x_2,m) \\ =&\begin{cases}\dfrac{(-1)^{-m+x_1}(\sqrt{\eta})^{x_2+m}(\sqrt{1-\eta})^{x_1-m}(x_1+x_2-m)!\,{}_2F_1(-m,-x_2;1-m+x_1;\frac{\eta-1}{\eta})}{(x_1-m)!x_2!} & ,(x_1+x_2\ge m)\&(x_1>m) \\ \dfrac{(\sqrt{\eta})^{-m+2x_1+x_2}(\sqrt{1-\eta})^{m-x_1}m!\,{}_2F_1(-x_1,m-x_1-x_2;1+m-x_1;\frac{\eta-1}{\eta})}{(m-x_1)!x_1!} & ,(x_1+x_2\ge m)\&(x_1\le m) \\ 0 & ,x_1+x_2<m\end{cases}.\end{aligned} \tag{131}$$

We can then define that,

$$v_i(m,x_1,x_2,y_1,y_2) = \vartheta(m,x_1,x_2)\frac{(\tanh r_F)^m\sqrt{x_1!}\sqrt{x_2!}}{\cosh r_F\sqrt{m!}\sqrt{(x_1+x_2-m)!}}\zeta_i(x_1+x_2-m,y_1,y_2). \tag{132}$$

We can obtain that,

$$|\delta_i\rangle = \sum_{m,x_1,x_2,y_1,y_2} v_i(m,x_1,x_2,y_1,y_2)\,|m,x_1,x_2,y_1,y_2\rangle\,, \tag{133}$$

$$\begin{aligned} Tr_{F_2F_1}(|\delta_i\rangle\langle\delta_i|) &= \sum_{m,x_1}(\langle m,x_1|\otimes I_{B_2E_1E_2})|\delta_i\rangle\langle\delta_i|(|m,x_1\rangle\otimes I_{B_2E_1E_2}) \\ &= \sum_{m,x_1,x_2,x_3,y_1,y_2,y_3,y_4} v_i(m,x_1,x_2,y_1,y_2)\bar{v}_i(m,x_1,x_3,y_3,y_4)|x_2,y_1,y_2\rangle\langle x_3,y_3,y_4| \\ &= \sum_{x_2,x_3,y_1,y_2,y_3,y_4}(\sum_{m,x_1} v_i(m,x_1,x_2,y_1,y_2)\bar{v}_i(m,x_1,x_3,y_3,y_4))|x_2,y_1,y_2\rangle\langle x_3,y_3,y_4|. \end{aligned} \tag{134}$$

We then define that,

$$\Phi_i(x_2,y_1,y_2,x_3,y_3,y_4) = \sum_{m,x_1} v_i(m,x_1,x_2,y_1,y_2)\bar{v}_i(m,x_1,x_3,y_3,y_4). \tag{135}$$

Then we can use equations (87-88), (103), (113), (116-117), (131-132) and (135) to calculate $\rho_{E_1E_2}^{x=\lambda_i}$. Similarly, we can use the equations (87-88), (113), (118-120), (131-132) and (135) to calculate $\rho_{E_1E_2}^{p=\lambda_i}$.

4. We final consider the situation that we use the heterodyne detection and consider the detection efficiency and the electrical noise. According to equations (122-123), we can obtain the density matrix of the state at $B_2E_1E_2F_2F_1$. Then, the state after passing through a beam splitter with transmissivity 0.5 can be given by,

$$\begin{aligned} \rho_{G_1B_3E_1E_2F_2F_1} =& (R_{G_0B_2}\otimes I_{E_1E_2F_2F_1})(|0\rangle\langle 0|\otimes\rho_{B_2E_1E_2F_2F_1})(R_{G_0B_2}^{\dagger}\otimes I_{E_1E_2F_2F_1}) \\ =& \sum_{i=0}^{N-1} p_i(R_{G_0B_2}\otimes I_{E_1E_2F_2F_1})(|0\rangle\langle 0|\otimes|\delta_i\rangle\langle\delta_i|)(R_{G_0B_2}^{\dagger}\otimes I_{E_1E_2F_2F_1}) \\ =& \sum_{i=0}^{N-1} p_i((R_{G_0B_2}\otimes I_{E_1E_2F_2F_1})(|0\rangle\otimes|\delta_i\rangle))((R_{G_0B_2}\otimes I_{E_1E_2F_2F_1})(|0\rangle\otimes|\delta_i\rangle))^{\dagger}. \end{aligned} \tag{136}$$

We define that,

$$|\ell_i\rangle_{G_1B_3E_1E_2F_2F_1} = |\ell_i\rangle = (R_{G_0B_2}\otimes I_{E_1E_2F_2F_1})(|0\rangle\otimes|\delta_i\rangle), \tag{137}$$

and we can further obtain that,

$$\rho_{B_3E_1E_2} = Tr_{G_1F_2F_1}(\rho_{G_1B_3E_1E_2F_2F_1}) = \sum_{i=0}^{N-1} p_i Tr_{G_1F_2F_1}(|\ell_i\rangle\langle\ell_i|). \tag{138}$$

Similar to the above derivations, we can use the equation (103) (where $\rho_{x_j,\Xi}^{E_1E_2} = (\langle x_j|\otimes I_{E_1E_2})\rho_{B_3E_1E_2}(|x_j\rangle\otimes I_{E_1E_2})$) to calculate $\rho_{E_1E_2}^{x=\lambda_i}$ with the higher calculating speed. So, we will start to calculate $\rho_{x_j,\Xi}^{E_1E_2}$ in this situation next. According to equations (87-88) and (131-132), we can get the expression of the $|\delta_i\rangle$ and can further obtain that,

$$|0\rangle\otimes|\delta_i\rangle = \sum_{m,x_1,x_2,y_1,y_2} v_i(m,x_1,x_2,y_1,y_2)\,|0,x_2,y_1,y_2,m,x_1\rangle\,. \tag{139}$$

We define $\hat{a}$ as the annihilation operator at G_0 and $\hat{b}$ as the annihilation operator at B_2, then we can obtain that,

$$|0\rangle\otimes|\delta_i\rangle = \sum_{y_1,y_2,m,x_1}(\sum_{x_2} v_i(m,x_1,x_2,y_1,y_2)\frac{(\hat{b}^{\dagger})^{x_1}}{\sqrt{x_2!}}\,|0,0\rangle)\otimes|y_1,y_2,m,x_1\rangle\,. \tag{140}$$

Then we can obtain that,

$$
\begin{aligned}
|\ell_i\rangle =&(R_{G_0B_2}\otimes I_{E_1E_2F_2F_1})(|0\rangle\otimes|\delta_i\rangle)\\
=&\sum_{y_1,y_2,m,x_1}(\sum_{x_2}v_i(m,x_1,x_2,y_1,y_2)/\sqrt{x_2!}(-\sqrt{0.5}\hat{a}^\dagger+\sqrt{0.5}\hat{b}^\dagger)^{x_2}|0,0\rangle)\otimes|y_1,y_2,m,x_1\rangle\\
=&\sum_{y_1,y_2,m,x_1}(\sum_{x_2}v_i(m,x_1,x_2,y_1,y_2)/\sqrt{x_2!}(\sum_{k=0}^{x_2}C_{x_2}^k(-\sqrt{0.5}\hat{a}^\dagger)^k(\sqrt{0.5}\hat{b}^\dagger)^{x_2-k})|0,0\rangle)\otimes|y_1,y_2,m,x_1\rangle\\
=&\sum_{y_1,y_2,m,x_1}(\sum_{x_2,k}v_i(m,x_1,x_2,y_1,y_2)/\sqrt{x_2!}\cdot C_{x_2}^k(-1)^k(\sqrt{0.5})^{x_2}(\hat{a}^\dagger)^k(\hat{b}^\dagger)^{x_2-k}|0,0\rangle)\otimes|y_1,y_2,m,x_1\rangle\\
=&\sum_{y_1,y_2,m,x_1}(\sum_{x_2,k}v_i(m,x_1,x_2,y_1,y_2)/\sqrt{x_2!}\cdot C_{x_2}^k(-1)^k(\sqrt{0.5})^{x_2}\sqrt{k!}\sqrt{(x_2-k)!}|k,x_2-k\rangle)\otimes|y_1,y_2,m,x_1\rangle\,.
\end{aligned}
\tag{141}
$$

Then we define $n_1=k$, $n_2=x_2-k$ and can further obtain that,

$$
\begin{aligned}
|\ell_i\rangle =&\sum_{n_1,n_2,y_1,y_2,m,x_1}v_i(m,x_1,n_1+n_2,y_1,y_2)/\sqrt{(n_1+n_2)!}C_{n_1+n_2}^{n_1}(-1)^{n_1}(\sqrt{0.5})^{n_1+n_2}\sqrt{n_1!}\sqrt{n_2!}|n_1,n_2,y_1,y_2,m,x_1\rangle\\
=&\sum_{n_1,x_2,y_1,y_2,m,x_1}v_i(m,x_1,n_1+x_2,y_1,y_2)/\sqrt{(n_1+x_2)!}C_{n_1+x_2}^{n_1}(-1)^{n_1}(\sqrt{0.5})^{n_1+x_2}\sqrt{n_1!}\sqrt{x_2!}|n_1,x_2,y_1,y_2,m,x_1\rangle\,.
\end{aligned}
\tag{142}
$$

We can further define that,

$$
\omega_i(n_1,x_2,y_1,y_2,m,x_1)=v_i(m,x_1,n_1+x_2,y_1,y_2)/\sqrt{(n_1+x_2)!}C_{n_1+x_2}^{n_1}(-1)^{n_1}(\sqrt{0.5})^{n_1+x_2}\sqrt{n_1!}\sqrt{x_2!}. \tag{143}
$$

We can obtain that,

$$
|\ell_i\rangle=\sum_{n_1,x_2,y_1,y_2,m,x_1}\omega_i(n_1,x_2,y_1,y_2,m,x_1)|n_1,x_2,y_1,y_2,m,x_1\rangle\,, \tag{144}
$$

$$
Tr_{G_1F_2F_1}(|\ell_i\rangle\langle\ell_i|)=\sum_{x_2,x_3,y_1,y_2,y_3,y_4}(\sum_{n_1,m,x_1}\omega_i(n_1,x_2,y_1,y_2,m,x_1)\bar{\omega}_i(n_1,x_3,y_3,y_4,m,x_1))|x_2,y_1,y_2\rangle\langle x_3,y_3,y_4|. \tag{145}
$$

We then define that,

$$
\Phi_i(x_2,y_1,y_2,x_3,y_3,y_4)=\sum_{n_1,m,x_1}\omega_i(n_1,x_2,y_1,y_2,m,x_1)\bar{\omega}_i(n_1,x_3,y_3,y_4,m,x_1). \tag{146}
$$

Then we can use equations (87-88), (103), (113), (116-117), (131-132), (143) and (146) to calculate $\rho_{E_1E_2}^{x=\lambda_j}$. Similarly, we can use equations (87-88), (113), (118-120), (131-132), (143) and (146) to calculate $\rho_{E_1E_2}^{p=\lambda_j}$.

Supplementary Note IV: Non-convex optimization method to calculate the leaked information under individual attacks

In this part, we introduce the non-convex optimization method[1] to calculate the leaked information under individual attacks, i.e., $\max_{\Pi} I(b;E,\Pi|\beta_y=m)$.

We can first define that,

$$
\rho_1=p(0_B|\beta_y=m)\rho_E^{x=m}, \tag{147}
$$

$$
\rho_2=p(1_B|\beta_y=m)\rho_E^{p=m}. \tag{148}
$$

Then, we define $\Pi=\{\Pi_k\}_{k=1,\dots,K}$ as a set of Eve's POVM. When under the sub-channel $\beta_y=m$ and Eve conducts the POVM Π, the joint probability p_{jk} of Bob encoding the key as j and Eve decoding the key as k, the marginal probability $p_{j\cdot}$ of Bob encoding the key as j, the marginal probability $p_{\cdot k}$ of Eve decoding the key as k and the accessible information between Bob and Eve $I(b;E,\Pi|\beta_y=m)$ are given by,

$$
p_{jk}=Tr(\rho_j\Pi_k), \tag{149}
$$

$$
p_{j\cdot}=\sum_k p_{jk},\, p_{\cdot k}=\sum_j p_{jk}, \tag{150}
$$

$$
I(b;E,\Pi|\beta_y=m)=\sum_{j,k}p_{jk}\log_2\frac{p_{jk}}{p_{j\cdot}p_{\cdot k}}. \tag{151}
$$

We can define that,

$$R_k = \sum_j \rho_j \log_2 \frac{p_{jk}}{p_{j\cdot} p_{\cdot k}}. \tag{152}$$

Since ρ_1 and ρ_2 are given, the accessible information between Bob and Eve, i.e. equation (151), is a nonlinear convex functional of Eve's POVM. The convexity,

$$I(b;E,\Pi^{(\lambda)}|\beta_y = m) \leq (1-\lambda)I(b;E,\Pi^{(1)}|\beta_y = m) + \lambda I(b;E,\Pi^{(2)}|\beta_y = m), \tag{153}$$

for $\Pi_k^{(\lambda)} = (1-\lambda)\Pi_k^{(1)} + \lambda\Pi_k^{(2)}$ with $0 \leq \lambda \leq 1$, is well known. It follows immediately from

$$(\frac{\partial}{\partial\lambda})^2 I(b;E,\Pi^{(\lambda)}|\beta_y = m) = \sum_{j,j',k} \frac{(p_{jk}^{(1)} p_{j'k}^{(2)} - p_{j'k}^{(1)} p_{jk}^{(2)})^2}{2p_{jk}^{(\lambda)} p_{j'k}^{(\lambda)} p_{\cdot k}^{(\lambda)}} \geq 0, \tag{154}$$

where $p_{jk}^{(\lambda)} = (1-\lambda)p_{jk}^{(1)} + \lambda p_{jk}^{(2)}$. So we obtain that $I(b;E,\Pi|\beta_y = m)$ is convex, it can achieve its global maximum value at the boundary of the convex set of all Eve's POVMs. Because we need to find the maximum value of $I(b;E,\Pi|\beta_y = m)$, i.e., find the maximum value of a convex function, we can use the non-convex optimization method to obtain the $\max_{\Pi} I(b;E,\Pi|\beta_y = m)$ and the best Eve's POVMs. And the convave optimization method is a steepest-ascent approach toward the maximum.

Then, the convex optimization algorithm is given by,

Step 1: Randomly generate a set of Eve's POVM Π.

Step 2: Calculate the p_{jk}, $p_{j\cdot}$, $p_{\cdot k}$ and R_k according to equations (147-150) and (152).

Step 3: Calculate the $G_k = I + a(R_k - \sum_k R_k \Pi_k)$ and $\widetilde{\Pi}_k = G_k^{\dagger}\Pi_k G_k$, where a is a positive constant value.

Step 4: Calculate $S = \sum_k \widetilde{\Pi}_k$ and update the Π_k with $S^{-1/2}\widetilde{\Pi}_k S^{-1/2}$. Update the $I(b;E,\Pi|\beta_y = m)$ according to equation (151). Back to *Step* 2 to start the next iteration round.

Here, we fix the total number of iteration rounds as 1000. The larger the value of a, the faster the convergence speed, which means the higher precision. However, if the a is too large, it will lead to the decrease of $I(b;E,\Pi|\beta_y = m)$ after one of iteration rounds. So, we need to choose a suitable constant value a according to the optimizing result. We also need to choose the number of elements K in the set of POVM Π. According to Ref.[2], we know that we never need more than $K = (rank(\rho_1 + \rho_2))^2$. Here we thus choose $K = (rank(\rho_1 + \rho_2))^2$.

Supplementary Note V: Proof of leaked information remaining unchanged for different purifications under collective attacks

In this part, we proof that the leaked information remains unchanged for different purifications under collective attacks.

Lemma 1.

$$Tr_A((X \otimes Y)M_{AB}(X^{\dagger} \otimes Y^{\dagger})) = Y(Tr_A((X \otimes I_B)M_{AB}(X^{\dagger} \otimes I_B)))Y^{\dagger}, \tag{155}$$

where X and Y is the square matrix at space A and B, respectively.

Proof.

$$\begin{aligned}
&Tr_A((X \otimes Y)M_{AB}(X^{\dagger} \otimes Y^{\dagger}))\\
=&\sum_i (((\langle i|_A \otimes I_B)(X \otimes Y)M_{AB}(X^{\dagger} \otimes Y^{\dagger})(|i\rangle_A \otimes I_B)) = \sum_i (((\langle i|_A \otimes I_B)(I_A \otimes Y)(X \otimes I_B)M_{AB}(X^{\dagger} \otimes Y_B)(I_A \otimes Y^{\dagger})(|i\rangle_A \otimes I_B))\\
=&\sum_i (((\langle i|_A \otimes Y)(X \otimes I_B)M_{AB}(X^{\dagger} \otimes I_B)(|i\rangle_A \otimes Y^{\dagger})) = \sum_i ((I_1 \otimes Y)(\langle i|_A \otimes I_B)(X \otimes I_B)M_{AB}(X^{\dagger} \otimes I_B)(|i\rangle_A \otimes I_B)(I_1 \otimes Y^{\dagger}))\\
=&\sum_i (Y(\langle i|_A \otimes I_B)(X \otimes I_B)M_{AB}(X^{\dagger} \otimes I_B)(|i\rangle_A \otimes I_B)Y^{\dagger}) = Y(\sum_i (((\langle i|_A \otimes I_B)(X \otimes I_B)M_{AB}(X^{\dagger} \otimes I_B)(|i\rangle_A \otimes I_B)))Y^{\dagger}\\
=&Y(Tr_A((X \otimes I_B)M_{AB}(X^{\dagger} \otimes I_B)))Y^{\dagger}.
\end{aligned} \tag{156}$$

□

Theorem 1. *We assume that $|ABE_1\rangle$ and $|ABE_2\rangle$ are two different purifications for ρ_{AB}, then we can obtain that,*

$$\chi_{bE_1}^{\beta_y=m} = \chi_{bE_2}^{\beta_y=m}, \tag{157}$$

i.e.,

$$\begin{aligned} &S(\rho_{E_1}^{\beta_y=m}) - p(0_B|\beta_y=m)S(\rho_{E_1}^{x=m}) - p(1_B|\beta_y=m)S(\rho_{E_1}^{p=m}) \\ =&S(\rho_{E_2}^{\beta_y=m}) - p(0_B|\beta_y=m)S(\rho_{E_2}^{x=m}) - p(1_B|\beta_y=m)S(\rho_{E_2}^{p=m}), \end{aligned} \tag{158}$$

where $\rho_{E_1}^{\beta_y=m} = p(0_B|\beta_y=m)\rho_{E_1}^{x=m} + p(1_B|\beta_y=m)\rho_{E_1}^{p=m}$ and $\rho_{E_2}^{\beta_y=m} = p(0_B|\beta_y=m)\rho_{E_2}^{x=m} + p(1_B|\beta_y=m)\rho_{E_2}^{p=m}$.

Proof. Because $|ABE_1\rangle$ and $|ABE_2\rangle$ are two different purifications for ρ_{AB}, we can obtain that,

$$\rho_{ABE_1} = |ABE_1\rangle\langle ABE_1|, \tag{159}$$

$$|ABE_2\rangle = (I_{AB}\otimes U_E)\,|ABE_1\rangle\,, \tag{160}$$

$$\rho_{ABE_2} = (I_{AB}\otimes U_E)\rho_{ABE_1}(I_{AB}\otimes U_E^\dagger), \tag{161}$$

where U_E is a unitary transformation. We define that $\hat{x}|x_m\rangle = m|x_m\rangle$, $\hat{p}|p_m\rangle = m|p_m\rangle$, $M_{AB}^x = I_A\otimes|x_m\rangle\langle x_m|$, and $M_{AB}^p = I_A\otimes|p_m\rangle\langle p_m|$. We can then obtain that,

$$\rho_{E_1}^{x=m} = \frac{Tr_{AB}((M_{AB}^x\otimes I_E)\rho_{ABE_1}((M_{AB}^x)^\dagger\otimes I_E))}{Tr(((M_{AB}^x)^\dagger\otimes I_E)(M_{AB}^x\otimes I_E)\rho_{ABE_1})}, \tag{162}$$

$$\begin{aligned} \rho_{E_2}^{x=m} =& \frac{Tr_{AB}((M_{AB}^x\otimes I_E)\rho_{ABE_2}((M_{AB}^x)^\dagger\otimes I_E))}{Tr(((M_{AB}^x)^\dagger\otimes I_E)(M_{AB}^x\otimes I_E)\rho_{ABE_2})} = \frac{Tr_{AB}((M_{AB}^x\otimes I_E)(I_{AB}\otimes U_E)\rho_{ABE_1}(I_{AB}\otimes U_E^\dagger)((M_{AB}^x)^\dagger\otimes I_E))}{Tr(((M_{AB}^x)^\dagger\otimes I_E)(M_{AB}^x\otimes I_E)(I_{AB}\otimes U_E)\rho_{ABE_1}(I_{AB}\otimes U_E^\dagger))} \\ =& \frac{Tr_{AB}((M_{AB}^x\otimes U_E)\rho_{ABE_1}((M_{AB}^x)^\dagger\otimes U_E^\dagger))}{Tr((I_{AB}\otimes U_E^\dagger)((M_{AB}^x)^\dagger\otimes I_E)(M_{AB}^x\otimes U_E)\rho_{ABE_1})} = \frac{Tr_{AB}((M_{AB}^x\otimes U_E)\rho_{ABE_1}((M_{AB}^x)^\dagger\otimes U_E^\dagger))}{Tr(((M_{AB}^x)^\dagger M_{AB}^x)\otimes(U_E^\dagger U_E)\rho_{ABE_1})} \\ =& \frac{Tr_{AB}((M_{AB}^x\otimes U_E)\rho_{ABE_1}((M_{AB}^x)^\dagger\otimes U_E^\dagger))}{Tr(((M_{AB}^x)^\dagger M_{AB}^x)\otimes(I_E I_E)\rho_{ABE_1})} = \frac{Tr_{AB}((M_{AB}^x\otimes U_E)\rho_{ABE_1}((M_{AB}^x)^\dagger\otimes U_E^\dagger))}{Tr(((M_{AB}^x)^\dagger\otimes I_E)(M_{AB}^x\otimes I_E)\rho_{ABE_1})}. \end{aligned} \tag{163}$$

According to Lemma 1 and equation (162), We further derive the equation (163) to obtain,

$$\rho_{E_2}^{x=m} = \frac{Tr_{AB}((M_{AB}^x\otimes U_E)\rho_{ABE_1}((M_{AB}^x)^\dagger\otimes U_E^\dagger))}{Tr(((M_{AB}^x)^\dagger\otimes I_E)(M_{AB}^x\otimes I_E)\rho_{ABE_1})} = U_E\frac{Tr_{AB}((M_{AB}^x\otimes I_E)\rho_{ABE_1}((M_{AB}^x)^\dagger\otimes I_E))}{Tr(((M_{AB}^x)^\dagger\otimes I_E)(M_{AB}^x\otimes I_E)\rho_{ABE_1})}U_E^\dagger = U_E\rho_{E_1}^{x=m}U_E^\dagger. \tag{164}$$

Similarly, we can get that,

$$\rho_{E_2}^{p=m} = U_E\rho_{E_1}^{p=m}U_E^\dagger. \tag{165}$$

Because $S(\bullet)$ is invariant under a unitary transformation (i.e., $S(\rho) = S(U\rho U^\dagger)$, where U is a unitary transformation), we can obtain that,

$$\begin{aligned} S(\rho_{E_2}^{\beta_y=m}) =&S(p(0_B|\beta_y=m)^{x=m}\rho_{E_2}^{x=m} + p(1_B|\beta_y=m)\rho_{E_2}^{p=m}) = S(p(0_B|\beta_y=m)U_E\rho_{E_1}^{x=m}U_E^\dagger + p(1_B|\beta_y=m)U_E\rho_{E_1}^{p=m}U_E^\dagger) \\ =&S(U_E(p(0_B|\beta_y=m)^{x=m}\rho_{E_1}^{x=m} + p(1_B|\beta_y=m)\rho_{E_1}^{p=m})U_E^\dagger) = S(U_E\rho_{E_1}^{\beta_y=m}U_E^\dagger) \\ =&S(\rho_{E_1}^{\beta_y=m}), \end{aligned} \tag{166}$$

$$S(\rho_{E_2}^{x=m}) = S(U_E\rho_{E_1}^{x=m}U_E^\dagger) = S(\rho_{E_1}^{x=m}), \tag{167}$$

$$S(\rho_{E_2}^{p=m}) = S(U_E\rho_{E_1}^{p=m}U_E^\dagger) = S(\rho_{E_1}^{p=m}). \tag{168}$$

Then we can easily get that,

$$\begin{aligned} &S(\rho_{E_1}^{\beta_y=m}) - p(0_B|\beta_y=m)S(\rho_{E_1}^{x=m}) - p(1_B|\beta_y=m)S(\rho_{E_1}^{p=m}) \\ =&S(\rho_{E_2}^{\beta_y=m}) - p(0_B|\beta_y=m)S(\rho_{E_2}^{x=m}) - p(1_B|\beta_y=m)S(\rho_{E_2}^{p=m}), \end{aligned} \tag{169}$$

i.e.,

$$\chi_{bE_1}^{\beta_y=m} = \chi_{bE_2}^{\beta_y=m}, \tag{170}$$

□

Thus, according to Theorem 1, we successfully proof that the leaked information remains unchanged for different purifications under collective attacks.

Supplementary Note VI: Simulation results of secret key rates for B/QPSK-BE-QKD when ignoring the imperfection of the detection

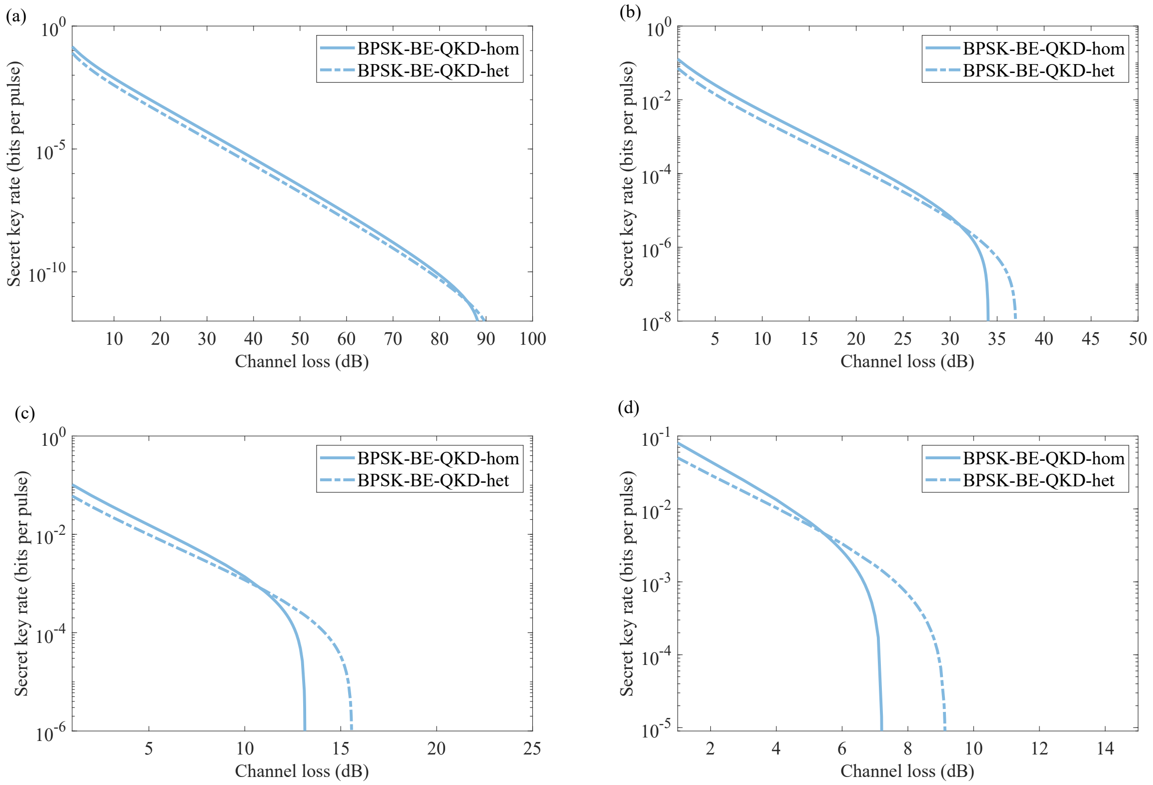

Supplementary Fig. 2. Secret key rates of BPSK-BE-QKD via the channel loss for the excess noise (a) $\varepsilon = 0.01$, (b) $\varepsilon = 0.02$, (c) $\varepsilon = 0.035$ or (d) $\varepsilon = 0.05$ in the linear Gaussian channel under collective attacks. Here we ignore the detection efficiency and the electrical noise. The modulation variance is $V_A = 0.5$ for BPSK-BE-QKD.

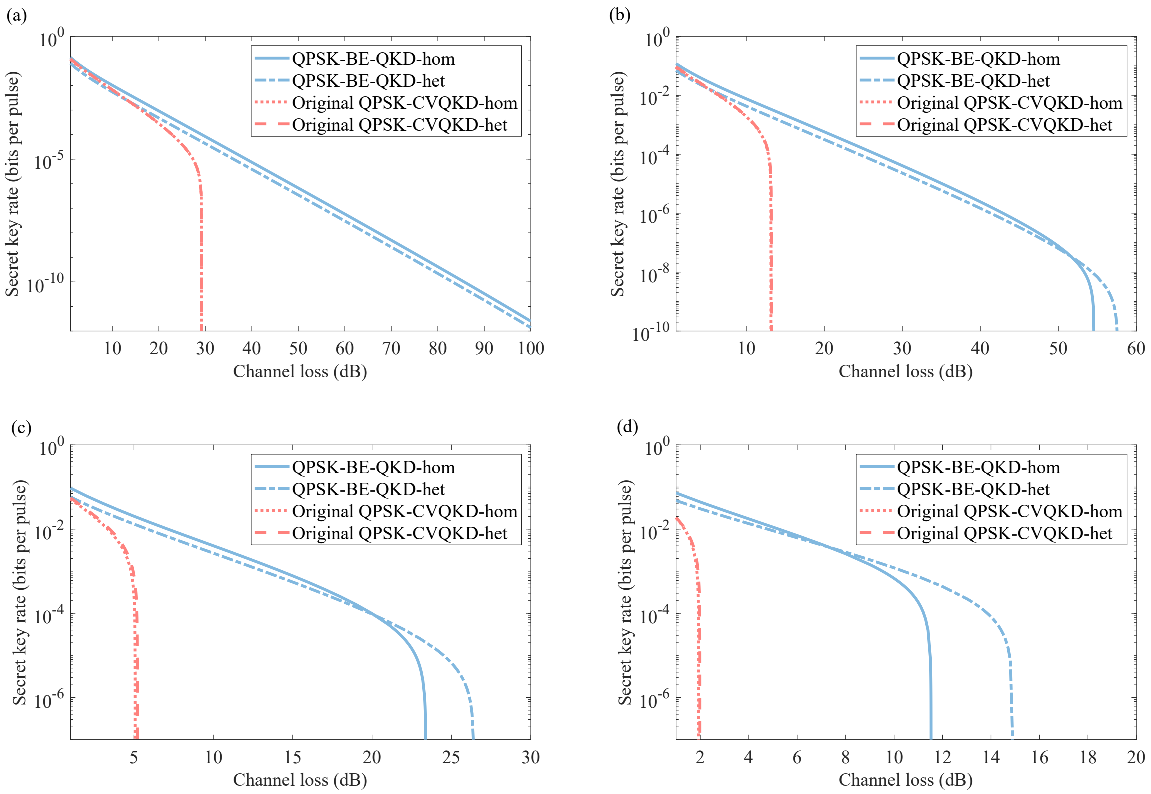

Supplementary Fig. 3. Secret key rates of QPSK-BE-QKD via the channel loss for the excess noise (a) $\varepsilon = 0.01$, (b) $\varepsilon = 0.02$, (c) $\varepsilon = 0.035$ or (d) $\varepsilon = 0.05$ in the linear Gaussian channel under collective attacks. Here we ignore the detection efficiency and the electrical noise. The modulation variance is $V_A = 1$ for QPSK-BE-QKD. For original QPSK-CVQKD, we set its modulation variance V_A to the optimal value obtained through traversal.

Supplementary Note VII: Simulation results of secret key rates for B/QPSK-BE-QKD when considering the imperfection of the detection

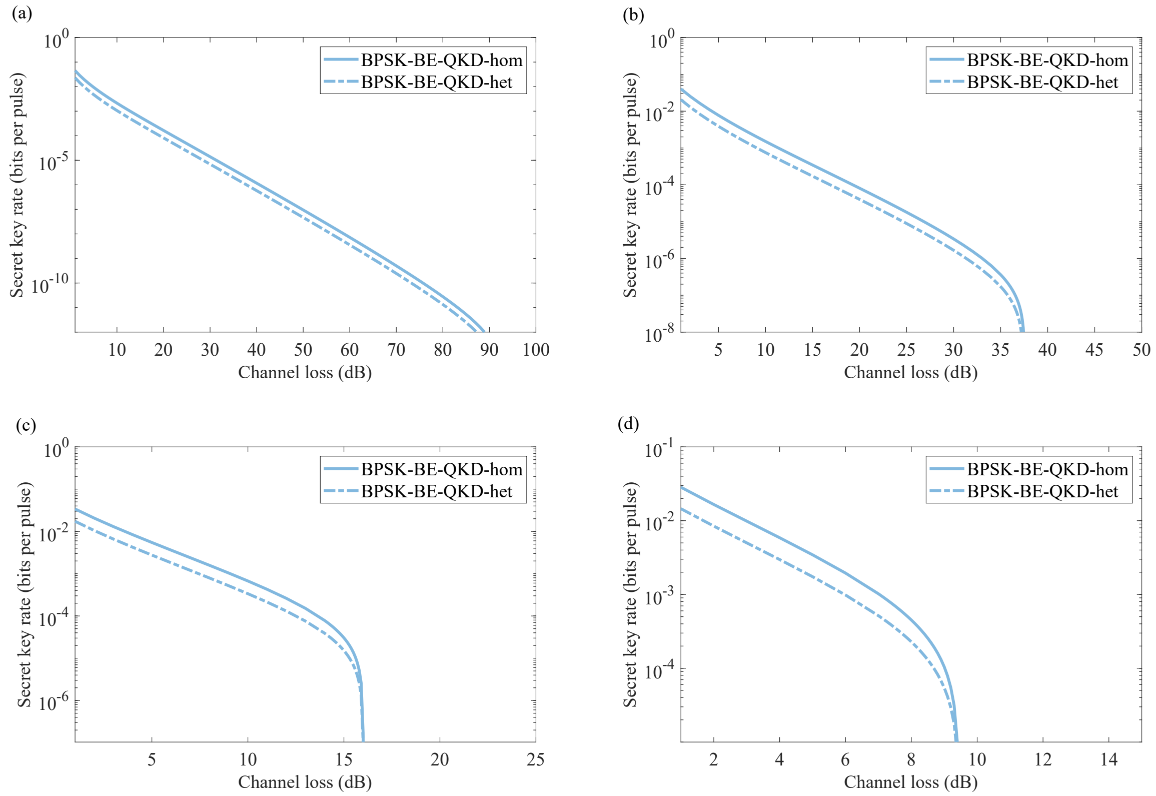

Supplementary Fig. 4. Secret key rates of BPSK-BE-QKD via the channel loss for the excess noise (a) $\varepsilon = 0.01$, (b) $\varepsilon = 0.02$, (c) $\varepsilon = 0.035$ or (d) $\varepsilon = 0.05$ in the linear Gaussian channel under collective attacks. Here the detection efficiency and the electrical noise are taken into account. The modulation variance is $V_A = 0.5$ for BPSK-BE-QKD. The detection efficiency and the electrical noise are set as $\eta = 0.375$ and $v_{el} = 0.4$, respectively.

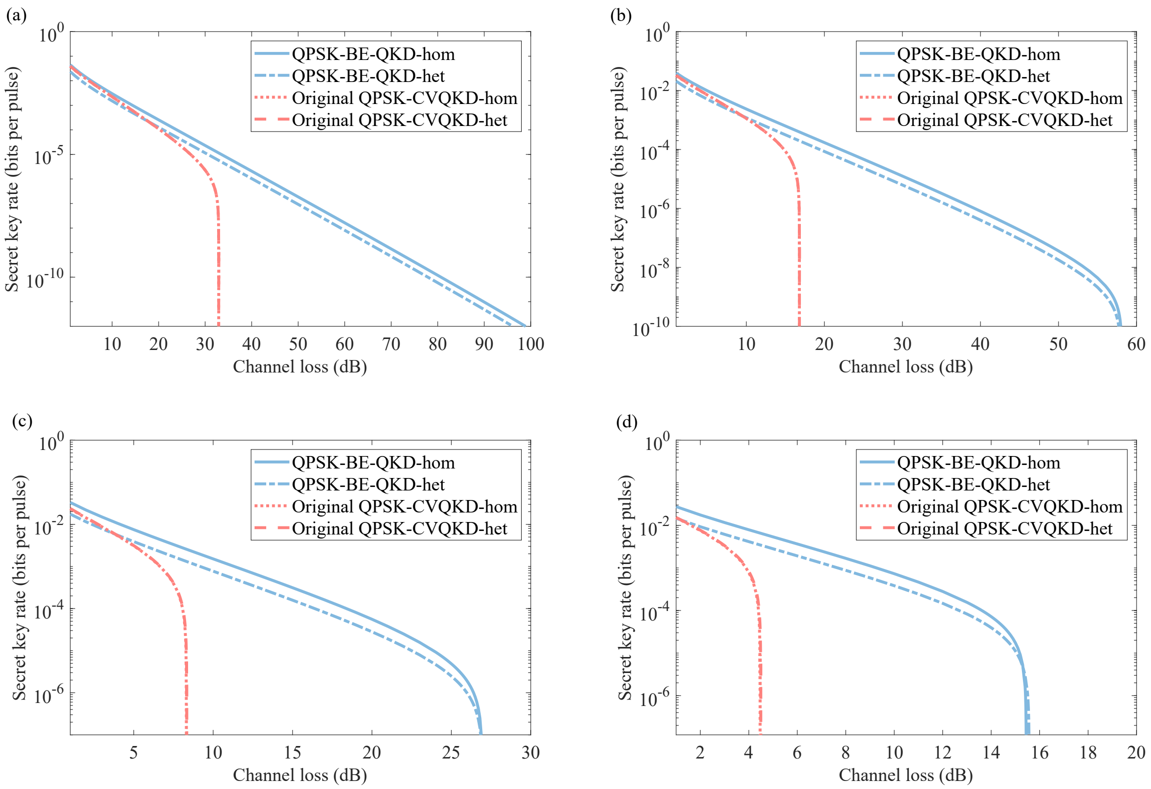

Supplementary Fig. 5. Secret key rates of QPSK-BE-QKD via the channel loss for the excess noise (a) $\varepsilon = 0.01$, (b) $\varepsilon = 0.02$, (c) $\varepsilon = 0.035$ or (d) $\varepsilon = 0.05$ in the linear Gaussian channel under collective attacks. Here the detection efficiency and the electrical noise are taken into account. The modulation variance is $V_A = 1$ for QPSK-BE-QKD. For original QPSK-CVQKD, we set its modulation variance V_A to the optimal value obtained through traversal. The detection efficiency and the electrical noise are set as $\eta = 0.375$ and $v_{el} = 0.4$, respectively.

Supplementary Note VIII: Simulation results of secret key rates for QPSK-BE-QKD under possible collective attacks partly traversed

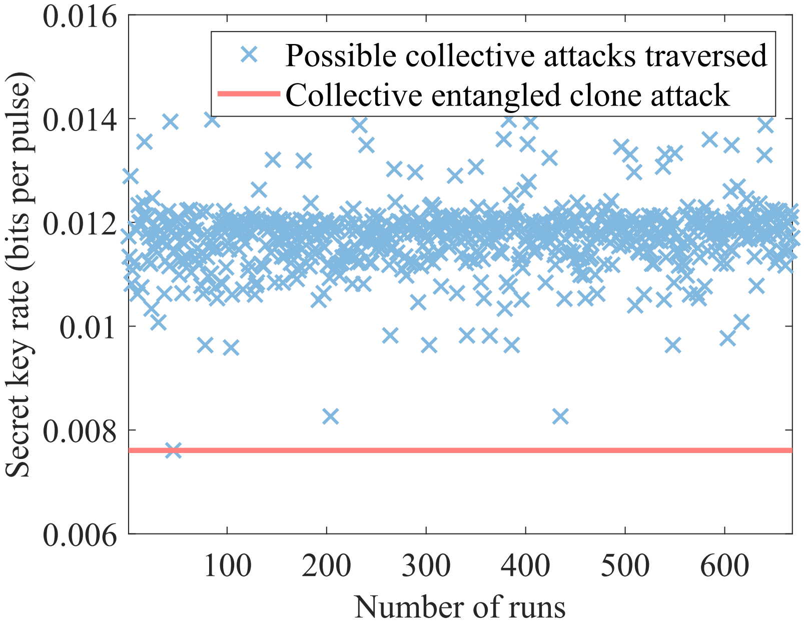

Supplementary Fig. 6. Secret key rates of QPSK-BE-QKD under possible collective attacks traversed when the modulation variance is $V_A = 1$, the channel loss is $T = 10$ dB and the excess noise is $\varepsilon = 0.02$. The blue points represent the secret key rate of QPSK-BE-QKD under possible collective attacks traversed and the red line represents the secret key rate of QPSK-BE-QKD under the collective entangling clone attack.

References

1. Řeháček J, Englert BG, Kaszlikowski D. Iterative procedure for computing accessible information in quantum communication. *Phys Rev A*. 2005;71(5):Article 054303.
2. Davies E. Information and quantum measurement. *IEEE Trans Inf Theory*. 1978;24(5):596-599.
